# Effects of exercise on depression and anxiety in postmenopausal women: a pairwise and network meta-analysis of randomized controlled trials

**DOI:** 10.1186/s12889-024-19348-2

**Published:** 2024-07-08

**Authors:** Bing Han, Yaya Duan, Peizhen Zhang, Liqing Zeng, Peng Pi, Jiping Chen, Guoli Du

**Affiliations:** 1 School of Sports Medicine and Rehabilitation, Beijing Sport University, Beijing, 100084 China; 2 Key Laboratory for Performance Training & Recovery of General Administration of Sport, Beijing Sport University, Beijing, 100084 China; 3 School of Physical Education, Shandong University, Jinan, Shandong 250061 China

**Keywords:** Exercise, Depression, Postmenopausal women, Pairwise meta-analysis, Network meta-analysis

## Abstract

**Background:**

Exercise has been identified as a promising non-pharmacological therapy for the management of depression, but there is still controversy over which type is most effective. We aimed to compare and rank the types of exercise that improve depression in postmenopausal women by quantifying information from randomized controlled trials (RCTs).

**Methods:**

The PubMed, Web of Science, SPORTDiscus, CNKI, The Cochrane Library, PsycINFO, EMBASE, and CINAHL Plus databases were searched to identify articles published from inception to 1 March 2024 reporting RCTs that examined the effectiveness of exercise on depression in postmenopausal women. The risk of bias was assessed using the revised Cochrane risk-of-bias tool for RCTs. The quality of the evidence for each comparison was graded using the online confidence in network meta-analysis tool (CINeMA). Standardized mean differences (SMDs) were calculated using the mean and standard deviation of pre-to-post intervention changes and then pooled using a random effects model in a pairwise meta-analysis using Review Manager 5.4. Then, a frequentist network meta-analysis was conducted using a random effects model was conducted to evaluate the efficacy of different exercise types using the network package of Stata 15.

**Results:**

This study included 26 studies involving 2,170 participants. The pairwise meta-analysis revealed that exercise had a significant positive effect on depression in postmenopausal women (SMD = -0.71, 95% confidence interval [CI] = -0.94 to -0.48; *I*^2^ = 78%). The network meta-analysis revealed that mind-body exercise (SMD = -0.97, 95% CI = -1.28 to -0.67), aerobic exercise (SMD = -0.58, 95% CI = -0.88 to -0.27) and multicomponent exercise (SMD = -0.57, 95% CI = -1.15 to -0.002) significantly reduced depression compared to the control intervention. Mind-body exercise had the highest probability of being the most effective intervention. Exercise interventions also showed positive effects on anxiety. Most studies were judged to have some concerns regarding their risk of bias, and the confidence in evidence was often very low according to CINeMA.

**Conclusion:**

For postmenopausal women, there is very low to moderate quality evidence that exercise interventions are an effective antidepressant therapy, with mind-body exercise most likely being the optimal type.

**Trial registration:**

This meta-analysis was prospectively registered with PROSPERO (registration number: CRD42024505425).

**Supplementary Information:**

The online version contains supplementary material available at 10.1186/s12889-024-19348-2.

## Introduction

Depression is acknowledged as a leading cause of disability worldwide [1, 2]. Current diagnostic guidelines and reports define it as a prolonged period of low mood or loss of pleasure or interest in activities [2], possibly accompanied by persistent symptoms such as insomnia, fatigue, reduced self-efficacy and suicidal tendencies [3]. It often causes substantial suffering to patients and places a significant burden on their families and society. Even subthreshold depression can result in distress or disability [4], and severe cases may lead to suicide [3].

Menopause is an inevitable and critical phase in the female life cycle [5]. While depression occurs in all age groups, its prevalence is notably higher in women during and after perimenopause [6, 7]. A large meta-analysis showed that the global prevalence of depression was 33.9% in perimenopausal women and 34.9% in postmenopausal women [8]. This increase is often attributed to hormonal fluctuations in estrogen and progesterone, sleep disturbances, and heightened stress susceptibility [4]. Ageing, emerging health issues and changes in family dynamics further contribute to depression [9]. Despite its significant impact on the health-related quality of life among postmenopausal women [10], the mental health concerns of this demographic remain largely unaddressed.

Common clinical treatments for depression typically involve medication or hormone therapy and behavioural interventions [11]. However, these interventions often have known and unknown side effects [12], limited accessibility and acceptance, and associated stigma [13]. The stigma associated with pharmacological and psychological interventions may deter many from seeking treatment [14]. Notably, about two-thirds of adults with depression do not receive adequate care [15], increasing to three-quarters in underdeveloped regions [16]. In addition, approximately one-third of patients with depression do not respond to multiple approved antidepressant medications [17]. Existing research indicates that postmenopausal women tend to respond less to antidepressants than younger women [18]. Moreover, pharmacological and psychological interventions often fail to address the physical comorbidities associated with depression [19]. Therefore, while conventional treatments still require significant attention and further research, there is a pressing need for more evidence-based treatments.

Exercise is often defined as an effective complement or alternative to drugs and psychotherapy [20], noted for its high accessibility, acceptability, and safety. Due to its extensive physiological and psychological benefits, the World Health Organization recommends it for diverse populations [21]. Research has shown that even physical activity below public health recommendations can yield significant mental health benefits [22]. A network meta-analysis of over 200 randomized controlled trials (RCTs) showed that some exercise interventions may have more potent antidepressant effects than selective serotonin reuptake inhibitors [20].

Given its benefits, several preliminary studies have explored the effects of exercise interventions in postmenopausal women [23–26]. Farzane et al. [24]. reported that eight weeks of pilates significantly improved depression in the studied population. Similarly, Gutiérrez et al. [25]. found that exercise could improve depressive symptoms in patients with moderate or severe depression. However, some studies have reported different findings. For example, Williams et al. [26]. reported that 12 months of group exercise did not significantly reduce depression in patients compared to controls. Additionally, Afonso et al. [23]. discovered that yoga could significantly improve patients’ depression, whereas stretching exercises (SE) could not.

The controversy over the effectiveness of exercise interventions may arise from several factors. Firstly, differences in the physiological mechanisms among exercise interventions can lead to varied effects on depression [20]. Secondly, the severity of depression may cause significant differences in outcomes [27]. For example, studies focusing on healthy postmenopausal women with lower baseline levels of depression may encounter a floor effect [28]. Additionally, the different control groups used across studies [29], some of which may positively impact mental health, further complicate comparisons.

Unfortunately, there is a dearth of research exploring the impact of exercise on depression among postmenopausal women. In addition, the potential moderating effects of baseline health status and intervention parameters in this context have not been sufficiently studied. Other concerns are whether improvements (assuming effectiveness) for postmenopausal women justify recommendation and whether the effects of various exercise interventions are comparable. The lack of comprehensive systematic reviews on the effects of exercise on depression in postmenopausal women impedes both understanding and the development of effective, evidence-based exercise prescriptions for this population.

In conclusion, there is an urgent need for a comprehensive systematic review assessing the effects of exercise on depression among postmenopausal women. Moreover, given the variability among exercise interventions and the inherent limitations in pairwise meta-analyses, conducting a network meta-analysis is essential to discern the differential effects of various types of exercise. Therefore, this study had two aims. First, to determine the efficacy of exercise interventions in reducing depression levels in postmenopausal women and assess whether this efficacy is influenced by the intervention duration and population characteristics. Second, to identify the optimal exercise intervention should the initial intervention prove effective.

## Methods

### Data source and search strategy

This systematic review and meta-analysis was prospectively registered with PROSPERO (registration number: CRD42024505425) and is reported according to the updated Preferred Reporting Items for Systematic Reviews and Meta-Analyses (PRISMA) statement [30] and the implementing PRISMA in Exercise, Rehabilitation, Sports Medicine and Sports Science (PERSiST) guidelines [31]. The PRISMA checklist is available in Supplemental Material 1.

The following online databases were searched from their inception to 1 March 2024: PubMed, Web of Science, SPORTDiscus, China National Knowledge Infrastructure (CNKI), The Cochrane Library, PsycINFO, EMBASE and CINAHL Plus. The search strategy was constructed using a combination of medical subject heading (MeSH) terms and their synonyms. The MeSH terms included postmenopause, exercise, depression and randomized controlled trial. The synonyms for each term were selected and added to the search strategy based on their frequency of occurrence in specific databases. Boolean logic operators were used to link these terms, and the search strategy was customised for each database’s specific requirements. The complete search strategy, applied across all databases, is detailed in Supplemental Material 2. Additionally, the references of meta-analyses on relevant topics were thoroughly searched to ensure the inclusion of all articles meeting the study criteria.

### Study selection and eligibility criteria

Articles identified in the electronic databases were imported into the EndNote software (version 20.0; Clarivate, Philadelphia, PA, USA) to facilitate the removal of duplicates. Next, two researchers (BH and YYD) independently assessed their titles and abstracts for potential inclusion. Then, they screened the full texts of relevant articles against the study’s specified inclusion and exclusion criteria to identify those to be included in the analyses. Any discrepancies in study selection between the two researchers were resolved through discussion.

The Population, Intervention, Comparison, Outcomes, and Study Design (PICOS) framework was used to structure the eligibility criteria. Studies eligible for this meta-analysis were RCTs (1) examining postmenopausal women without hormone therapy, any types of cancer, regular exercise habits or other comorbid conditions that may affect cognitive function; (2) examining at least one of the five exercise intervention arms of interest (SE, aerobic exercise [AE], resistance training [RT], mind-body exercise [MBE] and multicomponent exercise [ME]) implemented for at least four weeks; (3) including a non-exercise control group (e.g. usual care, wait-list control conditions or health education) or comparing two of the exercise interventions of interest (only included in the network meta-analysis); (4) examining the pre-post effects of exercise interventions on depression using a validated scale; and (5) published in English or Chinese.

### Outcomes

The primary outcome assessed in this study was depression, a common mental disorder. Since anxiety is a common comorbidity of depression and shares numerous symptoms with it [3, 32], we designated anxiety as a secondary outcome measure.

### Coding

The characteristics of the interventions were appropriately coded based on physiological mechanisms and patterns. This article categorised exercise into five broad types: SE, AE, RT, MBE, and ME. Detailed descriptions of each type of exercise intervention are provided in Supplemental Material 3. The duration of the interventions was coded in weeks or months, with six months being the threshold used to differentiate long-term and short-term interventions.

### Data extraction

Two researchers (BH and YYD) independently extracted the data. Any disagreements were resolved through discussion until a consensus was reached or by consulting a third researcher (PZZ). The following data were extracted: basic article information (first author, publication year, country, sample size for each arm and dropout rate), participant characteristics (age and health status), intervention parameters (exercise intensity, frequency, duration per session and overall length) and other information (comparator information, tools used to assess outcomes and reported outcomes [mean ± standard deviation (SD)]). For each outcome, we extracted the mean differences directly or calculated them based on the pre- and post-intervention mean ± SD and sample sizes for the meta-analyses. When relevant statistics were incompletely reported, we calculated the mean and SD using the sample size, standard error, confidence interval (CI), range and *p*-value. If the article lacked essential information, the corresponding author was contacted to acquire the necessary details.

### Risk of bias and certainty assessment

Two researchers (BH and YYD) used Version 2 of the Cochrane risk-of-bias tool [33](ROB 2.0) for RCTs to assess the risk of bias for all included studies. Any disagreements were resolved through discussion. The quality of the included studies was assessed based on six indicators: (1) randomization process bias, (2) deviations from intended interventions bias, (3) missing outcome data bias, (4) outcome measurement bias, (5) reported result selection bias and (6) overall bias. Each indicator has three risk levels: low, some concerns, and high. The overall bias of studies was classified as ‘low risk’ (all domains low risk), ‘some concerns’ (concerns in at least one domain but no high risks), or ‘high risk’ (high risk in at least one domain).

We used the Confidence in Network Meta-Analysis [34] (CINeMA) web tool to assess the quality of evidence for each comparison. It considers six factors that could lead to a downgrade: within-study bias, reporting bias, indirectness, imprecision, heterogeneity and incoherence. For within-study bias, comparisons were downgraded when most of the studies providing direct evidence for comparisons were ‘some concerns’ or ‘high risk’. For imprecision, we considered a ± 0.24 threshold for standardised mean differences (SMDs) as clinically meaningful [35]. For heterogeneity, CINeMA considered prediction intervals. Finally, the quality of evidence for each comparison was categorised as high, moderate, low, or very low.

### Statistical analysis

Pairwise and network meta-analyses were conducted using Review Manager (version 5.4; Cochrane, London, UK) or STATA (version 15.0; StataCorp, College Station, TX, USA) software. Since depression and anxiety measurements were continuous or sequential and measured on different scales, the effect estimates were represented as SMDs with 95% CIs. The statistical significance was set at *P* < 0.05.

In the pairwise meta-analysis, heterogeneity among studies was evaluated using the *I*^*2*^ statistic and the *Q* test. The recommendations of the Cochrane Group [36], state that an *I*^*2*^ of < 40% signifies low heterogeneity, 30–60% signifies moderate heterogeneity, 50–90% signifies substantial heterogeneity, and 75–100% signifies considerable heterogeneity. The *Q* and *I²* calculations can be found in Supplemental Material 4. A fixed-effects model was used when *I²* was ≤ 50%, and the random-effects model was used otherwise. Publication bias was assessed using visual interpretation of funnel plots and Egger tests. Once significant publication bias was detected, the trim-and-fill method was used to adjust the results, ensuring the robustness of our findings. In the subgroup analyses, participants’ age (< 60 and ≥ 60), health status (healthy, with depressive symptoms or other illnesses) and intervention duration (short-term and long-term) were assessed as categorical variables. Three types of sensitivity analyses were conducted: (1) the leave-one-out method; (2) the exclusion of studies that used health education as the control group, which enabled a clearer observation of the effects of exercise interventions; and (3) excluding studies judged to have an overall high risk of bias. Results that retained their significance after these analyses were deemed robust.

The network meta-analysis was performed according to the current operation guidelines [37] and based on the frequentist framework. It was performed with the following steps. First, a network geometry was created to explore the comparative relationships between several exercise interventions and controls. Second, global inconsistency tests were performed to statistically evaluate whether the network meta-analysis’s consistency assumptions were met. Local inconsistency was assessed using the node-splitting method. When a closed loop was formed among interventions, a loop inconsistency test was conducted to assess the consistency between direct and indirect evidence within that loop. Third, a predictive interval plot was created to illustrate the direct comparative effects of various exercise interventions. Fourth, the interventions were ranked to determine their superiority based on the surface under the cumulative ranking curve (SUCRA) and mean rank. The SUCRA offline area map was plotted; the larger the area under the curve or SUCRA value (0–100%), the more effective the intervention in reducing depression. Finally, sensitivity analyses were conducted, excluding studies judged to have an overall high risk of bias.

## Results

### Identification and screening of studies

**Fig. 1:**
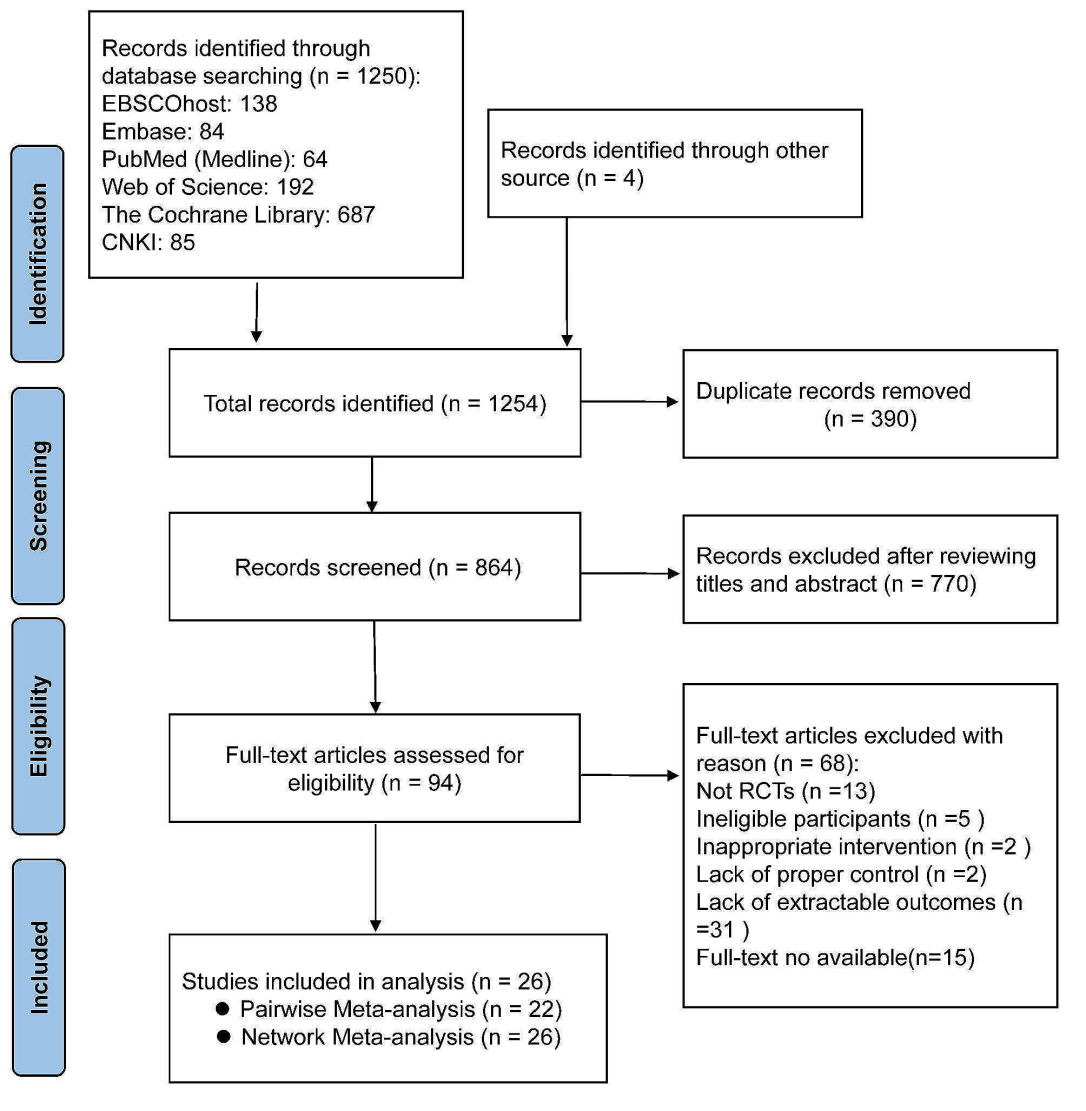
Flowchart of the screening process

A total of 1,250 articles related to the research topic were retrieved from the online databases, and an additional four were identified from the reference lists of previous reviews. After removing duplicate studies using EndNote (*n* = 390), 864 eligible articles were identified. Following the initial screening of titles and abstracts, 94 articles were considered potentially relevant. Subsequently, 68 (Supplemental Material 5) that did not meet this study’s inclusion and exclusion criteria were excluded, resulting in the final inclusion of 22 studies in the pairwise meta-analysis and 26 in the network meta-analysis. The detailed study selection process is shown in Fig. 1.

### Studies’ characteristics

The quantitative analysis included the data of 2,170 participants (1,425 in the treatment group and 745 in the control group) from 26 RCTs [23–26, 38–59]. The selected RCTs originated from 11 countries. Among the 26 studies, 22 compared the effects of the exercise and control interventions, and four compared the effects of various types of exercises.

The included RCTs examined the effects of different exercise interventions: two (100 participants) examined the effects of SE, 12 (657 participants) examined the effects of AE, four (220 participants) examined the effects of RT, 12 (334 participants) examined the effects of MBE, and three (114 participants) examined the effects of ME.

The participant characteristics differed across the studies included in this meta-analysis. Twelve studies targeted healthy postmenopausal women, four focused on postmenopausal women with depressive symptoms, and eight examined postmenopausal women with other health conditions, including obesity and heart failure. The mean age of participants ranged from 52.5 [56] to 80.4 [40] years. The most common tool for measuring depression was the Geriatric Depression Scale (*n* = 6) [25, 40, 45, 46, 52, 53], followed by the Beck Depression Inventory (*n* = 5) [23, 41, 47, 51, 56] and Hospital Anxiety and Depression Scale (*n* = 4) [24, 38, 39, 43], while the remaining studies used various scales. The studies’ detailed characteristics are provided in Supplemental Material 6.

### Results of risk of bias and certainty assessment

**Fig. 2:**
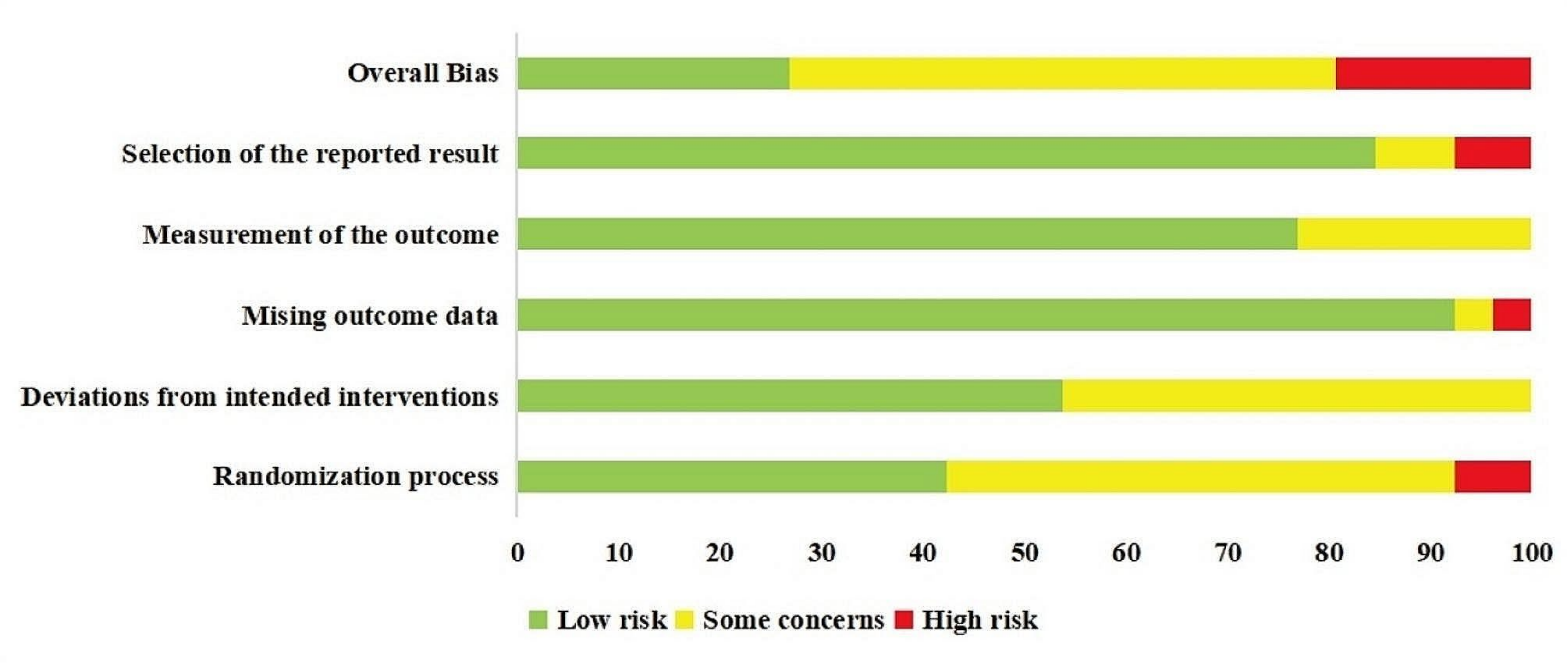
Summary of the risk of bias in the included RCTs

Regarding the risk of bias in the included RCTs, most were judged to have some concerns (*n* = 14, 53.8%), followed by low risk (*n* = 7, 26.9%) and high risk (*n* = 5, 19.2%). Most RCTs implemented and adequately described randomization procedures. The main source of bias was the inadequate reporting of allocation concealment and blinding of assessors. Supplemental Material 7 provides the risk of bias ratings for each study. The risk of bias assessment is summarised in Fig. 2.

Supplemental Material 8 shows the strength of evidence for each network estimate. Overall, the comparison between MBE and the control intervention had moderate confidence, while the other comparisons had very low confidence. The main reasons for downgrading were imprecision and heterogeneity, primarily due to the broad CIs and prediction intervals. Furthermore, all comparisons had some concerns regarding within-study bias. Reporting bias was also difficult to robustly assess because a direct comparison was often only provided in fewer than 10 studies. No studies showed problematic incoherence, meaning the direct and indirect evidence agreed.

### Pairwise meta-analysis

**Fig. 3:**
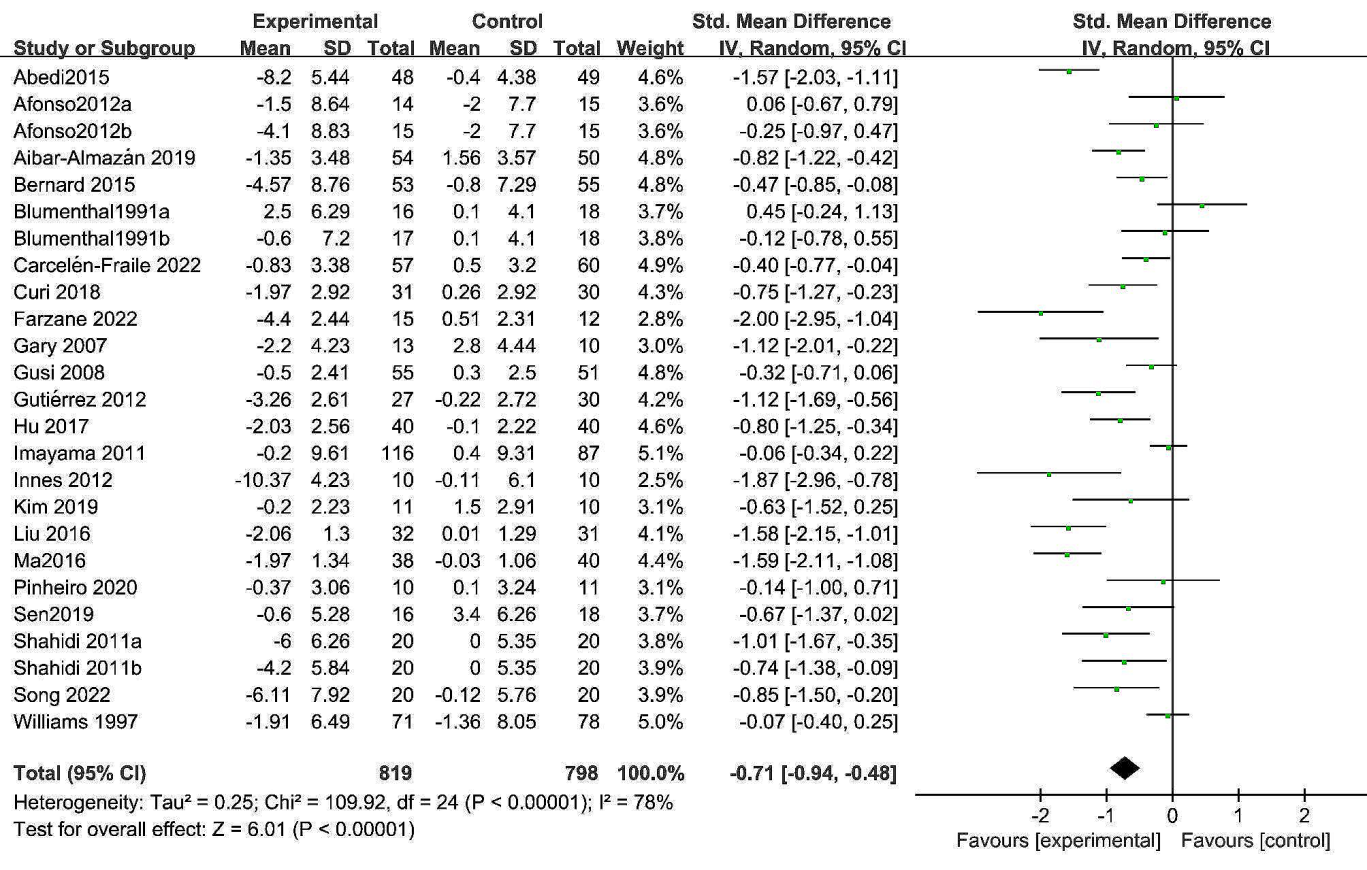
Forest plot of the effects of exercise training on depression

Twenty-five comparisons from 22 RCTs involving 1,564 participants (exercise group = 819, control group = 745) were eligible for pairwise meta-analysis on depression. Exercise interventions demonstrated a clinically significant positive effect on depression in postmenopausal women (SMD = -0.71, 95% CI = -0.94 to -0.48, *P* < 0.001; Fig. 3), with an overall *I*^*2*^ of 78%. The funnel plot appeared slightly asymmetric, and Egger’s test indicated the presence of slight publication bias (*P* = 0.047; Supplemental Material 9.1.1). The trim-and-fill method was used to account for potential unpublished studies. The results showed that no additional studies were needed to balance the funnel plot, indicating strong stability of the outcomes (Supplemental Material 9.1.2).

The subgroup analyses (Table 1) revealed that exercise interventions significantly reduced depression levels in postmenopausal women, regardless of intervention duration, age, and health status. However, their effect size varied across different subgroups, with short-term interventions (SMD = -0.85, 95% CI = -1.16 to -0.55; *I*^*2*^ = 75%) proving more effective than long-term interventions (SMD = -0.39, 95% CI = -0.61 to -0.16; *I*^*2*^ = 55%), suggesting that intervention duration may be one source of heterogeneity. In addition, those with depressive symptoms gained greater health benefits (SMD = -0.95, 95% CI = -1.44 to -0.45; *I*^*2*^ = 78%). Moreover, those in the early postmenopause (*n* = 7; SMD = -0.70, 95% CI = -1.24 to -0.16; *I*^*2*^ = 85%) and late postmenopause (*n* = 18; SMD = -0.71, 95% CI = -0.97 to -0.45; *I*^*2*^ = 75%) derived similar benefits from exercise interventions. These results remained unchanged after three types of sensitivity analysis (Supplemental Material 9.2): a leave-one-out approach, excluding studies with healthy education as the control group (SMD = -0.67, 95% CI = -0.93 to -0.42, *P* < 0.001; *I*^*2*^ = 80%) or excluding studies judged to have an overall high risk of bias (SMD = -0.81, 95% CI = -1.06 to -0.55, *P* < 0.001; *I*^*2*^ = 80%).

**Table 1:** The subgroup analyses for the primary outcomes

Outcomes	Subgroup	*N*	Z	SMD	95%CI	I^2^ (%)	*P*	Weight
Duration of intervention	Short-term	17	5.53	-0.85	-1.16,-0.55	75	< 0.001	64.4
	Long-term	8	3.38	-0.39	-0.61,-0.16	55	< 0.001	35.6
	Test for subgroup differences: Chi² = 5.88, *P* = 0.02, *I²* = 83%							
Health status	Healthy	12	4.77	-0.78	-1.09,-0.46	76	< 0.001	49.1
	DS	5	3.74	-0.95	-1.44,-0.45	78	< 0.001	21.2
	OI	8	2.17	-0.36	-0.69,-0.04	58	0.02	29.6
	Test for subgroup differences: Chi² = 4.97, *P* = 0.08, *I²* = 59.8%							
Age	EPM	7	2.53	-0.70	-1.24,-0.16	85	< 0.001	27.6
	LPM	18	5.42	-0.71	-0.97,-0.45	75	< 0.001	72.4
	Test for subgroup differences: Chi² = 0.00, *P* = 0.97, *I²* = 0%							

*DS: depressive symptoms; OI: other Illnesses; EPM: early postmenopausal; LPM: late postmenopausal

### Network meta-analysis

**Fig. 4:**
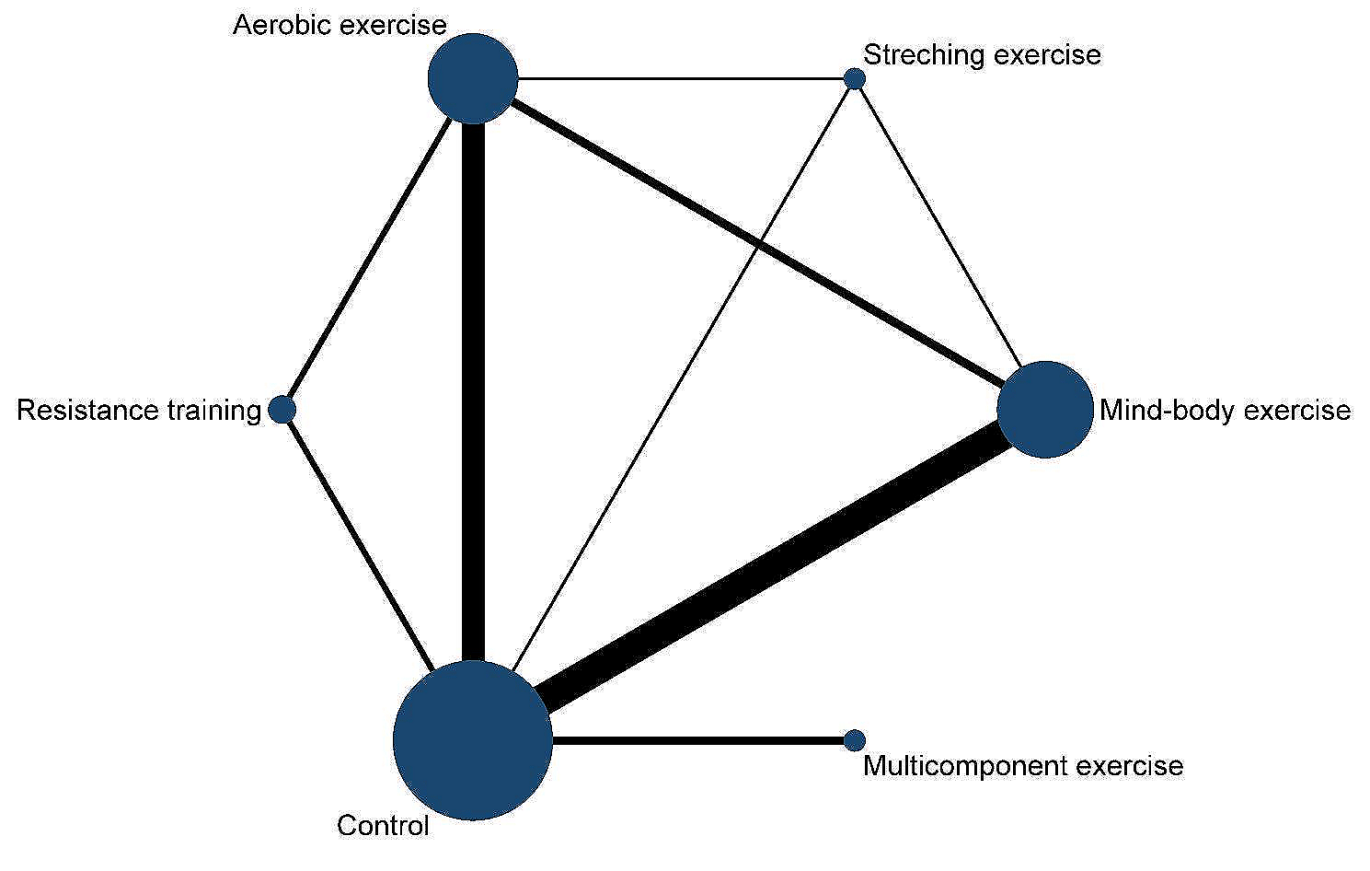
Network plot of depression comparisons in the network meta-analysis

Twenty-six RCTs involving 2,170 patients (exercise group = 1,425, control group = 745) were eligible for the network meta-analysis. Figure 4 shows the network of pairwise comparisons across all RCTs. Inconsistency test based on network analysis revealed no significant global or local inconsistency (*P* > 0.05), indicating that the consistency assumption could be accepted at the overall level. Within the depression network, the loop inconsistency test did not indicate inconsistency (inconsistency factor (IF) = 0.10 to 0.67) or loop-specific heterogeneity (τ^2^ < 0.001). The network meta-analysis results revealed that, in both global and specific closed-loop contexts, direct and indirect evidence does not differ significantly (Supplemental Material 9.3).

**Fig. 5:**
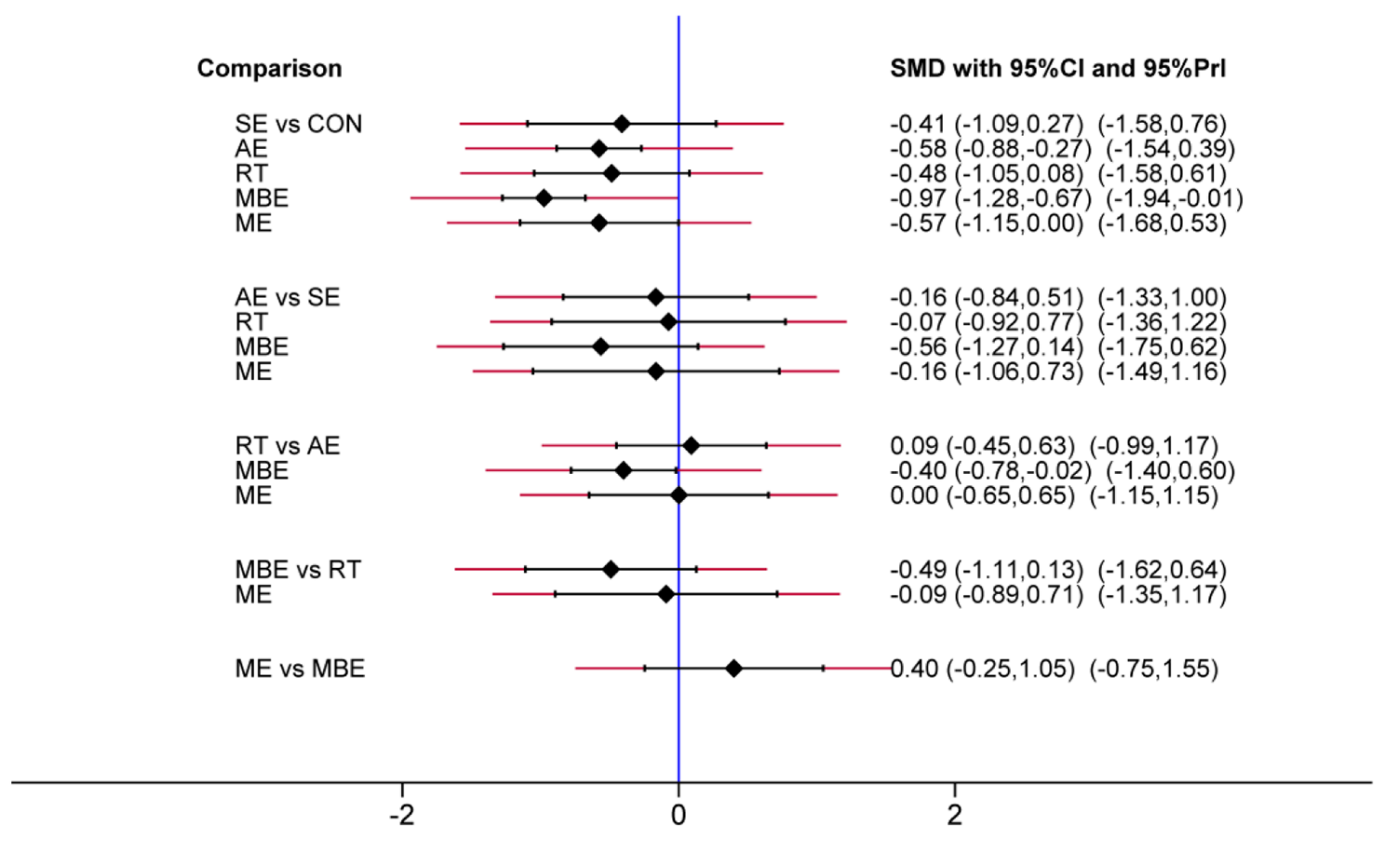
Predictive interval plot of network meta-analysis for depression. Abbreviations: CON: control; SE: stretching exercise; AE: aerobic exercise; RT: resistance training; MBE: mind-body exercise; ME: multicomponent exercise

**Fig. 6:**
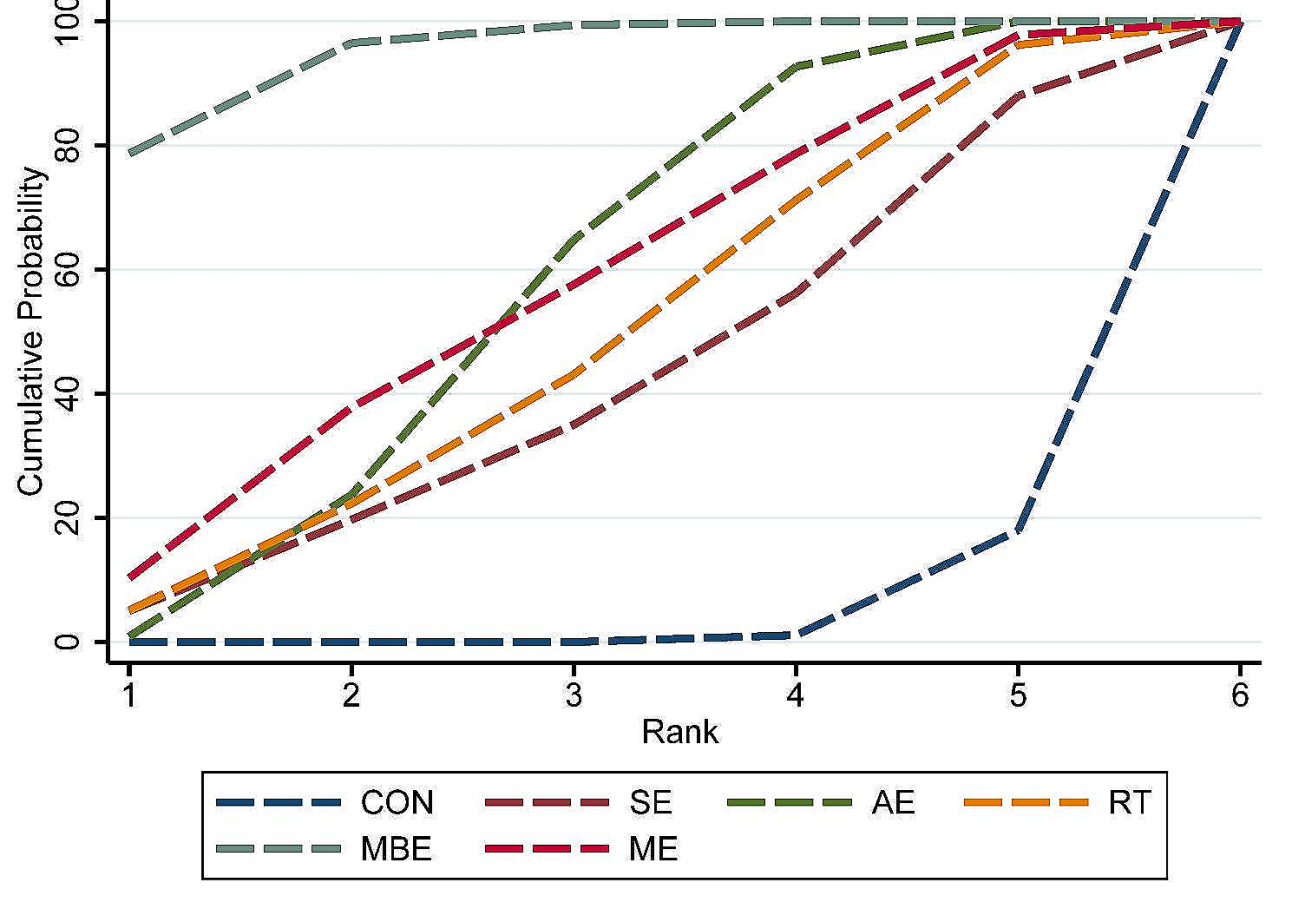
Cumulative ranking probability plots for depression. Abbreviations: CON: control; SE: stretching exercise; AE: aerobic exercise; RT: resistance training; MBE: mind-body exercise; ME: multicomponent exercise

The predictive interval plot (Fig. 5) shows the pairwise comparison outcomes among interventions and their predictive intervals. It indicated that only MBE (SMD = -0.97, 95% CI = -1.28 to -0.67), AE (SMD = -0.58, 95% CI = -0.88 to -0.27) and ME (SMD = -0.57, 95% CI = -1.15 to -0.002) significantly reduced depression compared to the control intervention. The pairwise comparisons between exercise interventions indicated that MBE were significantly more effective than AE (SMD = -0.40, 95% CI = -0.78 to -0.02). Figure 6 shows the rankings of the exercise interventions based on the cumulative probability plots and SUCRA. MBE had the highest probability (SUCRA = 94.7%; Supplemental Material 9.4) of being the most effective exercise type, followed by AE and ME. The sensitivity analysis results showed that when five studies judged to be at high risk of bias were excluded, MBE remained the best intervention (SUCRA = 95.7%; Supplemental Material 9.5).

### Secondary outcomes

For anxiety, 13 RCTs involving 1,076 patients (exercise group = 578, control group = 498) were found to be eligible for pairwise meta-analysis. The results revealed that exercise interventions positively affected anxiety (SMD = -0.53, 95% CI = -0.72 to -0.33, *P* < 0.001; *I*^*2*^ = 57.0%). The network meta-analysis included 15 RCTs with 1,285 participants. The global inconsistency test indicated no significant global inconsistency in the results (*P* > 0.05). Within the anxiety network, the loop inconsistency test did not indicate high inconsistency (IF = 0.05 to 0.30) or loop-specific heterogeneity (τ^2^ < 0.001). The results showed that AE (SMD = -0.34, 95% CI = -0.63 to -0.05) and MBE (SMD = -0.69, 95% CI = -0.92 to -0.47) significantly reduced anxiety in postmenopausal women compared to the control intervention, with MBE having the highest probability (SUCRA = 95.4%) of being the most effective exercise type. The specific results of the meta-analysis for anxiety are presented in Supplemental Material 10.

## Discussions

### Main findings

This meta-analysis included 26 RCTs with 2,170 participants. To our knowledge, this is the first pairwise and network meta-analysis to investigate the effects of exercise on depression in postmenopausal women, including a ranking of five common exercise interventions. This study contributes to the ongoing debate about the efficacy of exercise interventions in reducing depression in this demographic, confirming that exercise significantly reduces depression. Among the five evaluated exercise interventions, only MBE, AE and ME significantly reduced depression. MBE emerged as the most likely optimal type for alleviating depression in postmenopausal women. Furthermore, subgroup analyses demonstrated that exercise interventions significantly reduced depression in postmenopausal women, irrespective of their duration, participants’ age, or their health status. The subgroup analysis and network meta-analysis results indicated that the intervention’s duration and exercise type may be sources of heterogeneity. In addition, we found that only MBE and AE significantly improved anxiety, with MBE showing the largest effect. The sensitivity analysis showed robust results for both pairwise and network meta-analysis.

### Effect of exercise on depression in postmenopausal women

Similar to other studies in diverse populations [20, 27, 60–62], our study demonstrated that exercise interventions significantly reduced depression levels in postmenopausal women. Given that the minimum clinically significant difference established in earlier study were − 0.24 (SMD) for patients with major depressive disorders [35], the change observed here may be considered clinically significant. The robustness of our pairwise meta-analysis is supported by three sensitivity analyses. The antidepressant mechanisms of exercise are manifold and, while not fully elucidated, can be categorized into biological and psychosocial mechanisms [9]. Regarding biological mechanisms, exercise potentially alleviates depression by enhancing neuroplasticity [63], promoting anti-inflammatory responses [64], regulating oxidative stress [63], and modulating the neuroendocrine system, including attenuating the hypothalamic-pituitary-adrenal axis response to stress [65]. Psychosocial mechanisms appear to be more varied. Research suggests that exercise improves mental health by enhancing self-esteem [47], self-efficacy [9], social support [66], body image and social interaction [22].

Our subgroup analysis of the primary outcomes revealed several significant phenomena or conclusions that warrant further discussion. Firstly, short-term interventions reduced depression to a greater extent than long-term interventions, consistent with numerous previous studies [27, 29, 61]. A possible explanation is that some long-term control groups exhibit a placebo effect [41], and participation in long-term experiments increases social interaction [26]. Alternatively, longer experimental periods often have higher dropout rates, which can dilute the overall effect [61], especially with intention-to-treat analysis [48]. Another possible explanation is that the effect of exercise on depression may indeed wane over time. Secondly, patients with depression derive greater benefits from exercise, potentially due to the lower baseline level of depression in healthy populations, which illustrating a floor effect. Thirdly, our findings indicate that exercise interventions provide similar benefits across different age stages in postmenopausal women. This consistency may arise from significant changes, such as hormonal fluctuations and shifts in social roles, occurring primarily during menopause [5]. Once this critical phase is passed, age appears to have a minimal effect on the efficacy of exercise interventions for depression. However, it is crucial to recognize that the age categorisations based on the average age in study reports may not adequately reflect the diversity of ages; therefore, caution is advised when interpreting these results.

MBE focuses on promoting the unity of mind, body, and behaviour and is characterised by gentle and slow movements [67]. The network meta-analysis results showed that MBE, ME and AE significantly reduced depression in postmenopausal women, while the other two types did not. The CIs of the effect sizes for SE, RT, and ME compared to the control intervention were relatively broad, likely due to the limited number of studies examining them. MBE may be the most suitable form of exercise intervention to reduce depression in postmenopausal women, consistent with findings in elderly populations [62, 68]. This preference may arise from the gentler nature of MBE, which postmenopausal women often favour. Unlike other exercise forms, MBE uniquely foster interoceptive states [69], potentially explaining their superior antidepressant effects. Furthermore, given that the intensity of MBE and AE is generally lower, it is notable that when exercise intensities are comparable, some exercise modalities [68] may be more effective in alleviating depression in postmenopausal women and potentially in a wider demographic.

### Effect of exercise on anxiety in postmenopausal women

Anxiety is a common comorbidity of depression, and not only do they share some similar symptoms, but they may also have a causal relationship [32]. Our study found that exercise interventions could have a moderate mitigating effect on the anxiety of postmenopausal women. Individuals with anxiety tend to withdraw from social situations, and engaging in exercise represents a change in social behavior [70]. Exercise interventions can also exert anti-anxiety effects by eliciting specific physiological responses that may mimic sensations associated with anxiety or panic, such as a rapid heartbeat [71]. Some potential mechanisms through which exercise may combat depression (e.g. enhancing self-efficacy through progressive, positive feedback [9]) are also used to explain its anti-anxiety effects. Additionally, our network meta-analysis found that MBE had the highest probability of being the most effective exercise type for alleviating anxiety, further supporting our recommendation of MBE.

### Implications for clinical practice and future research

This pairwise and network meta-analysis provides valuable insights and recommendations for clinical professionals and researchers. Most importantly, it confirms the significant antidepressant effects of exercise interventions in postmenopausal women, highlighting the particular efficacy of MBE. These should be considered complementary therapies in clinical settings due to their significant benefits for depression and anxiety. Notably, consistent with prior research [27, 29, 61], we observed that long-term interventions did not yield as favourable results as short-term interventions. While several hypotheses are proposed to explain this phenomenon, a detailed investigation falls outside the scope of this review and should be addressed in future research through multiple follow-up points and rigorously controlled RCTs. Despite our findings supporting the superior efficacy of MBE, it is crucial to emphasise that the value of other exercise interventions remains undiminished. In order to enhance adherence in clinical settings, postmenopausal women should be given the choice of their preferred exercise type, although MBE will likely be the most favoured. Additionally, while our study categorised and analysed different types of exercises, the actual interventions within categories may still vary substantially. Therefore, when sufficient studies are published, future research should further classify exercise intervention methods.

### Strengths and limitations

Our study had several strengths. To our knowledge, it is the first meta-analysis to examine the efficacy of exercise interventions in reducing depression in postmenopausal women. By extending standard meta-analyses with network meta-analyses, we could determine the most effective types of exercise interventions, enhancing recommendations for clinical practice. Moreover, the robustness of our findings is supported by multiple sensitivity and subgroup analyses, which also helped to identify potential sources of heterogeneity. Lastly, using the ROB 2.0 and CINeMA online tools enabled us to effectively assess the risk of bias and the certainty of the evidence in the included studies.

However, our study also had some limitations. Firstly, heterogeneity was difficult to avoid due to the experimental design (e.g. different exercise intervention modalities) used in the included studies. To enhance the reliability of our findings, we conducted subgroup and sensitivity analyses across multiple dimensions. Moreover, we used network meta-analysis to rank and compare exercise intervention modalities, identifying them as a likely significant source of heterogeneity. Secondly, the diverse scales used to measure depression may have also contributed to heterogeneity, although we prioritised the most commonly used scales. Thirdly, the predominant pre-post intervention design of the included studies limited our analysis to data extracted solely from intervention endpoints due to the absence of long-term follow-up data. Fourthly, while we have retrieved as many relevant articles as possible, our inclusion of grey literature may still have deficiencies. Lastly, since fewer original studies had examined SE, RT, and ME, their effect sizes had broader CIs. Consequently, results should be interpreted with caution. Future research should include larger samples to more thoroughly assess the efficacy of these exercise interventions.

## Conclusions

This pairwise and network meta-analysis synthesised the findings of 26 RCTs. It highlighted the efficacy of exercise in postmenopausal women and provided some key findings about exercise therapy for clinical professionals and researchers. Exercise interventions have a significant effect in reducing depression levels in postmenopausal women, with MBE likely being the best form of exercise intervention. The meta-analysis for anxiety also supports our recommendation for exercise, particularly MBE. Future research should develop more acceptable exercise intervention models and conduct additional multi-arm RCTs to provide robust direct evidence.

### Electronic supplementary material

Below is the link to the electronic supplementary material.

Supplementary Material 1

## Data Availability

All data generated or analyzed during this study are included in this published article and its supplementary information files.

## Abbreviations

AE: Aerobic exercise
CI: Confidence interval
CINeMA: Confidence in Network Meta-Analysis
IF: Inconsistency factors
MBE: Mind-body exercise
ME: Multicomponent exercise
PERSiST: Prisma in Exercise, Rehabilitation, Sport medicine and Sports science
PRISMA: Preferred Reporting Items for Systematic Reviews and Meta-Analyses
RCT: Randomized controlled trials
ROB 2.0: Version 2 of the Cochrane risk-of-bias tool
RT: Resistance training
SD: Standard deviation
SE: Stretching exercise
SMD: Standardized mean difference
SUCRA: Surface under the cumulative ranking curve
WHO: World Health Organization
