# Supplementary material for: Effects of exercise on depression and anxiety in postmenopausal women: a pairwise and network meta-analysis of randomized controlled trials
Source: BMC Public Health. 2024 Jul 8;24:1816. doi: 10.1186/s12889-024-19348-2 (PMC11229230; doi:10.1186/s12889-024-19348-2)
Supplement: Supplementary file 1 — Supplementary Material 1 [file 12889_2024_19348_MOESM1_ESM.docx]

Effects of exercise on depression and anxiety in postmenopausal women: a pairwise and network meta-analysis of randomized controlled trials

Context

[Supplemental Material 1 PRISMA 2020 checklist 2](#_Toc2642)

[Supplemental Material 2 Search Strategy 6](#_Toc25343)

[Supplemental Material 3 The Classifications of Exercise Interventions 9](#_Toc13016)

[Supplemental Material 4 The calculations of Q and I² 10](#_Toc21786)

[Supplemental Material 5 Description of Studies Excluded at Full Text Stage 12](#_Toc15496)

[Supplemental Material 6 Study Characteristics 17](#_Toc4836)

[Supplemental Material 7 The risk of bias for each study 22](#_Toc13818)

[Supplemental Material 8 Evaluation of the Certainty of evidence 23](#_Toc21856)

[Supplemental Material 9 The Analysis for Primary Outcomes 25](#_Toc13515)

[Supplemental Material 10 The Analysis for Anxiety 2](#_Toc29282)9

Supplemental Material 1 PRISMA 2020 checklist

Table 1 PRISMA 2020 checklist

| **Section and Topic** | **Item #** | **Checklist item** | **Location where item is reported** |
| --- | --- | --- | --- |
| **TITLE** | | |  |
| Title | 1 | Identify the report as a systematic review. | Title |
| **ABSTRACT** | | |  |
| Abstract | 2 | See the PRISMA 2020 for Abstracts checklist. | Abstract |
| **INTRODUCTION** | | |  |
| Rationale | 3 | Describe the rationale for the review in the context of existing knowledge. | Intro, para 7 |
| Objectives | 4 | Provide an explicit statement of the objective(s) or question(s) the review addresses. | Intro, para 8 |
| **METHODS** | | |  |
| Eligibility criteria | 5 | Specify the inclusion and exclusion criteria for the review and how studies were grouped for the syntheses. | Methods, 2.2. para 2 |
| Information sources | 6 | Specify all databases, registers, websites, organisations, reference lists and other sources searched or consulted to identify studies. Specify the date when each source was last searched or consulted. | Methods, 2.1.para 1-2 |
| Search strategy | 7 | Present the full search strategies for all databases, registers and websites, including any filters and limits used. | Supplemental Material 2 |
| Selection process | 8 | Specify the methods used to decide whether a study met the inclusion criteria of the review, including how many reviewers screened each record and each report retrieved, whether they worked independently, and if applicable, details of automation tools used in the process. | Methods, 2.2. para 1 |
| Data collection process | 9 | Specify the methods used to collect data from reports, including how many reviewers collected data from each report, whether they worked independently, any processes for obtaining or confirming data from study investigators, and if applicable, details of automation tools used in the process. | Methods, 2.5. |
| Data items | 10a | List and define all outcomes for which data were sought. Specify whether all results that were compatible with each outcome domain in each study were sought (e.g. for all measures, time points, analyses), and if not, the methods used to decide which results to collect. | Methods, 2.3. |
|  | 10b | List and define all other variables for which data were sought (e.g. participant and intervention characteristics, funding sources). Describe any assumptions made about any missing or unclear information. | Methods, 2.2. para 2 |
| Study risk of bias assessment | 11 | Specify the methods used to assess risk of bias in the included studies, including details of the tool(s) used, how many reviewers assessed each study and whether they worked independently, and if applicable, details of automation tools used in the process. | Methods, 2.5. |
| Effect measures | 12 | Specify for each outcome the effect measure(s) (e.g. risk ratio, mean difference) used in the synthesis or presentation of results. | Methods,  2.7. para 1 |
| Synthesis methods | 13a | Describe the processes used to decide which studies were eligible for each synthesis (e.g. tabulating the study intervention characteristics and comparing against the planned groups for each synthesis (item #5)). | Methods, 2.2. para 2 |
|  | 13b | Describe any methods required to prepare the data for presentation or synthesis, such as handling of missing summary statistics, or data conversions. | Methods, 2.5. |
|  | 13c | Describe any methods used to tabulate or visually display results of individual studies and syntheses. | Methods,2.7. |
|  | 13d | Describe any methods used to synthesize results and provide a rationale for the choice(s). If meta-analysis was performed, describe the model(s), method(s) to identify the presence and extent of statistical heterogeneity, and software package(s) used. | Methods,2.7. |
|  | 13e | Describe any methods used to explore possible causes of heterogeneity among study results (e.g. subgroup analysis, meta-regression). | Methods,  2.7. para 2 |
|  | 13f | Describe any sensitivity analyses conducted to assess robustness of the synthesized results. | Methods,  2.7. 2-3 |
| Reporting bias assessment | 14 | Describe any methods used to assess risk of bias due to missing results in a synthesis (arising from reporting biases). | Methods,  2.6. para 1 |
| Certainty assessment | 15 | Describe any methods used to assess certainty (or confidence) in the body of evidence for an outcome. | Methods,  2.6. para 2 |
| **RESULTS** | | |  |
| Study selection | 16a | Describe the results of the search and selection process, from the number of records identified in the search to the number of studies included in the review, ideally using a flow diagram. | Results, Figure 1 |
|  | 16b | Cite studies that might appear to meet the inclusion criteria, but which were excluded, and explain why they were excluded. | Supplemental Material 5 |
| Study characteristics | 17 | Cite each included study and present its characteristics. | Results, 3.2. |
| Risk of bias in studies | 18 | Present assessments of risk of bias for each included study. | Supplemental Material 7 |
| Results of individual studies | 19 | For all outcomes, present, for each study: (a) summary statistics for each group (where appropriate) and (b) an effect estimate and its precision (e.g. confidence/credible interval), ideally using structured tables or plots. | Results, 3.4.,3.5.,3.6. |
| Results of syntheses | 20a | For each synthesis, briefly summarise the characteristics and risk of bias among contributing studies. | Results, 3.3. |
|  | 20b | Present results of all statistical syntheses conducted. If meta-analysis was done, present for each the summary estimate and its precision (e.g. confidence/credible interval) and measures of statistical heterogeneity. If comparing groups, describe the direction of the effect. | Results, 3.4.,3.5.,3.6. |
|  | 20c | Present results of all investigations of possible causes of heterogeneity among study results. | Results, 3.4.,3.6. |
|  | 20d | Present results of all sensitivity analyses conducted to assess the robustness of the synthesized results. | Results, 3.4.,3.5. |
| Reporting biases | 21 | Present assessments of risk of bias due to missing results (arising from reporting biases) for each synthesis assessed. | Supplemental Material 9.1 |
| Certainty of evidence | 22 | Present assessments of certainty (or confidence) in the body of evidence for each outcome assessed. | Supplemental Material 8 |
| **DISCUSSION** | | |  |
| Discussion | 23a | Provide a general interpretation of the results in the context of other evidence. | Disc. 4.1. |
|  | 23b | Discuss any limitations of the evidence included in the review. | Disc. 4.5. |
|  | 23c | Discuss any limitations of the review processes used. | Disc. 4.5. |
|  | 23d | Discuss implications of the results for practice, policy, and future research. | Disc. 4.4. |
| **OTHER INFORMATION** | | |  |
| Registration and protocol | 24a | Provide registration information for the review, including register name and registration number, or state that the review was not registered. | abstract |
|  | 24b | Indicate where the review protocol can be accessed, or state that a protocol was not prepared. | NA |
|  | 24c | Describe and explain any amendments to information provided at registration or in the protocol. | NA |
| Support | 25 | Describe sources of financial or non-financial support for the review, and the role of the funders or sponsors in the review. | Funding |
| Competing interests | 26 | Declare any competing interests of review authors. | Competing interests |
| Availability of data, code and other materials | 27 | Report which of the following are publicly available and where they can be found: template data collection forms; data extracted from included studies; data used for all analyses; analytic code; any other materials used in the review. | Data availability |

Supplemental Material 2 Search Strategy

Table 2 Search strategy in Pubmed

| Step | Search strategy |
| --- | --- |
| #1 | "postmenopause"[mh] OR “post menopausal period”[tiab] OR postmenopausal[tiab] OR “Post Menopause”[tiab] OR “Senior woman”[tiab] OR “Elderly woman”[tiab] OR “Older woman”[tiab] 77347 |
| #2 | "exercise"[mh] OR exercises[tiab] OR “physical activity”[tiab] OR “aerobic exercise”[tiab] OR sport*[tiab] OR walk*[tiab] OR swim*[tiab] OR yoga[tiab] OR qigong[tiab] OR baduanjin[tiab] OR pilate[tiab] OR taichi[tiab] OR resistance[tiab] OR training[tiab] 2020893 |
| #3 | "depression"[mh] OR depress*[tiab] 614,375 |
| #4 | "randomized controlled trial"[pt] OR "controlled clinical trial"[pt] OR "randomized controlled trials as topic"[mh] OR "clinical trials as topic"[mh] OR "controlled clinical trials as topic"[mh] OR "clinical trial"[pt] OR "random allocation"[mh] OR randomized[tiab] OR randomised[tiab] OR randomization[tiab] OR randomly allocated[tiab] OR RCT[tiab] OR clinical trial*[tiab] OR clinical stud*[tiab] 2,081,133 |
| #5 | #1 AND #2 AND #3 AND #4 64 |

Table 3 Search strategy in Embase

| Step | Search strategy |
| --- | --- |
| #1 | ‘postmenopause’/exp OR ‘post menopausal’:ab,ti OR postmenopausal:ab,ti OR ‘Senior woman’:ab,ti OR ‘Elderly woman’:ab,ti OR ‘Older woman’:ab,ti 130,203 |
| #2 | ‘exercise’/exp OR exercises:ab,ti OR ‘physical activity’:ab,ti OR sport*:ab,ti OR ‘aerobic exercise’:ab,ti OR walk*:ab,ti OR qigong:ab,ti OR swim*:ab,ti OR yoga:ab,ti OR baduanjin:ab,ti OR pilate:ab,ti OR taichi:ab,ti OR resistance:ab,ti OR training:ab,ti 2,695,556 |
| #3 | ‘depression’/exp OR depress*:ab,ti 1,012,471 |
| #4 | 'randomized controlled trial'/exp OR randomized:ab,ti OR randomised:ab,ti OR randomization:ab,ti OR randomly:ab,ti OR RCT:ab,ti OR 'controlled clinical trial'/exp OR 'controlled clinical':ab,ti 1,972,600 |
| #5 | #1 AND #2 AND #5 141 |
| #6 | #1 AND #2 AND #5 AND ([article]/lim) 84 |

Table 4 Search strategy in The Cochrane Library

| Step | Search strategy |
| --- | --- |
| #1 | MeSH descriptor: [postmenopause] explode all trees 6,099 |
| #2 | (postmenopause OR postmenopausal OR “post menopausal” OR Senior woman OR Elderly woman OR Older woman):ti,ab,kw 25,647 |
| #3 | MeSH descriptor: [exercise] explode all trees 38,461 |
| #4 | (exercise* OR “physical activity” OR “aerobic exercise” OR sport* OR walk* OR swim* OR yoga OR qigong OR baduanjin OR pilate OR taichi OR resistance OR training):ti,ab,kw 314,383 |
| #5 | MeSH descriptor: [depression] explode all trees 105,473 |
| #6 | (depression OR depressive):ti,ab,kw 109,195 |
| #7 | #1 OR #2 26,157 |
| #8 | #3 OR #4 317,319 |
| #9 | #5 OR #6 146,186 |
| #10 | #7 AND #8 AND #9 ([trial]/lim) 529 |

Table 5 Search strategy in Web of Science

| Step | Search strategy |
| --- | --- |
| #1 | TS=("postmenopause" OR “post menopausal period” OR postmenopausal OR “Post Menopause” OR “Senior woman” OR “Elderly woman” OR “Older woman”) 136,880 |
| #2 | TS=("exercise" OR exercises OR “physical activity” OR “aerobic exercise” OR sport* OR walk* OR swim* OR yoga OR qigong OR baduanjin OR pilate OR taichi OR resistance OR training) 6,195,675 |
| #3 | TS=(depression OR depressive) 1,037,732 |
| #4 | TS=("randomized controlled trial" OR "controlled clinical trial" OR "clinical trial" OR "random allocation" OR randomized OR randomised OR randomization OR RCT) 1,769,072 |
| #5 | #1 AND #2 AND #3 AND #4 236 |
| #6 | #1 AND #2 AND #3 AND #4 NOT Review Article (Exclude – Document Types) 192 |

Table 6 Search strategy in EBSCOhost

| Step | Search strategy |
| --- | --- |
| #1 | AB=("postmenopause" OR “post menopausal period” OR postmenopausal OR “Post Menopause” OR Senior woman OR Elderly woman OR Older woman) |
| #2 | AB=("exercise" OR exercises OR “physical activity” OR “aerobic exercise” OR sport* OR walk* OR swim* OR yoga OR qigong OR baduanjin OR pilate OR taichi OR resistance OR training) |
| #3 | AB=(depression OR depressive) |
| #4 | AB=("randomized controlled trial" OR "controlled clinical trial" OR "clinical trial" OR "random allocation" OR randomized OR randomised OR randomization OR RCT) |
| #5 | #1 AND #2 AND #3 AND #4 138 |

*Note:* SPORTDiscus, PsycINFO and CINAHL Plus are all accessible through EBSCOhost for searching.

Table 7 Search strategy in CNKI

| #1 | 运动 + 锻炼 + 抗阻 + 八段锦 + 气功 + 六字诀 + 瑜伽 + 太极 + 步行 + 游泳 + 普拉提 2,,080,299 |
| --- | --- |
| #2 | 绝经后 + 老年女性 34,889 |
| #3 | 抑郁 231,181 |
| #4 | #1 AND #2 AND #3 85 |

Supplemental Material 3 The Classifications of Exercise Interventions

Table 8 The classifications of exercise interventions

| Types | Define |
| --- | --- |
| Aerobic exercise | Aerobic exercise aiming to improve cardiovascular fitness including walking, running, swimming or cycling. |
| Resistance training^[1]^ | A type of strength-building exercise program that requires the body muscle to exert a force against some form of resistance, such as weight, stretch bands, water, or immovable objects. Resistance exercise is a combination of static and dynamic contractions involving shortening and lengthening of skeletal muscles. |
| Stretching exercise^[2]^ | Stretching exercises are physical activities that aim to increase the flexibility of muscles and joints, which can enhance overall movement and decrease the risk of injuries. There are vaious types of stretching technigues including active, passive (relaxed), static, dynamic (gentle), ballistic (forced), isometric, and others. |
| Mind-body  Exercise^[3]^ | Mind-body exercise, aiming to improve participants’ mind-body coordination and awareness by practicing a series of controlled movements and focusing on interactions among the brain, body, mind, and behavior, such as Tachi, yoga, Qigong, pilates, baduanjin and dance. |
| Multicomponent exercise | A combination of at least 2 types of exercise, such as aerobic exercise, resistance training, and stretching exercise. |

**The above relates to the following references:**

1. Pubmed, Resistance Training,https://www.ncbi.nlm.nih.gov/mesh/?term=Resistance+training
2. Pubmed, Muscle Stretching Exercises, https://www.ncbi.nlm.nih.gov/mesh/?term=Muscle+Stretching+Exercises
3. Ives J C, Sosnoff J. Beyond the mind-body exercise hype.[J]. The Physician and sportsmedicine, 2000,28(3):67-81.

**Supplemental Material 4 The calculations of Q and I²**

**COMPUTING Q**

The first step in partitioning the variation is to compute Q, defined as


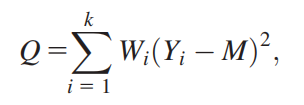


where Wi is the study weight (1/Vi), Yi is the study effect size, and M is the summary effect and k is the number of studies. In words, we compute the deviation of each effect size from the mean, square it, weight this by the inverse-variance for that study, and sum these values over all studies to yield the weighted sum of squares (WSS), or Q.

The same formula can be written as


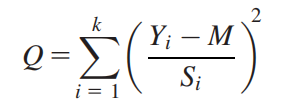


to highlight the fact that Q is a standardized measure, which means that it is not affected by the metric of the effect size index. The analogy would be to the standardized mean difference d, where the mean difference is divided by the within-study standard deviation.

Finally, an equivalent formula, useful for computations, is


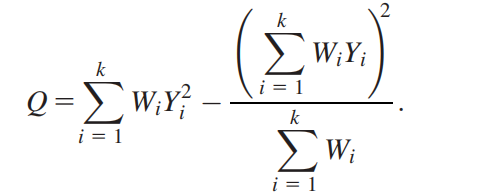


**THE I^2^ STATISTIC**


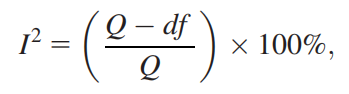


In this equation, Q is the Chi2 statistic and df is its degrees of freedom. I^2^ describes the percentage of the variability in effect estimates that is due to heterogeneity rather than sampling error (chance).

The statistic I^2^ can be viewed as a statistic of the form


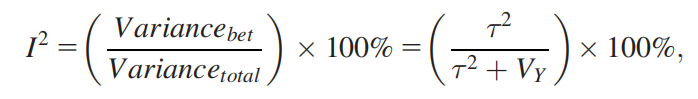


that is, the ratio of true heterogeneity to total variance across the observed effect estimates. However, this is not a true definition of I^2^ because in reality there is not a single V_Y_, since the within-study variances vary from study to study. The I^2^ statistic is a descriptive statistic and not an estimate of any underlying quantity.

**Explanation of I^2^**

Thresholds for the interpretation of the I^2^ statistic can be misleading, since the importance of inconsistency depends on several factors. A rough guide to interpretation in the context of meta-analyses of randomized trials is as follows:

- 0% to 40%: might not be important;
- 30% to 60%: may represent moderate heterogeneity*;
- 50% to 90%: may represent substantial heterogeneity*;
- 75% to 100%: considerable heterogeneity*.

**The above relates to the following references:**

[1] Michael Borenstein, Larry V. Hedges, Julian P. T. Higgins, et al. Introduction to Meta-Analysis (Chapter 16). Chichester, West Sussex, UK: John Wiley & Sons, 2009.

[2] Higgins JPT, Thomas J, Chandler J, Cumpston M, Li T, Page MJ, Welch VA (editors). Cochrane Handbook for Systematic Reviews of Interventions version 6.4 (Chapter 10.10). Cochrane, 2023. Available from www.training.cochrane.org/handbook.

Supplemental Material 5 Description of Studies Excluded at Full Text Stage

Table 9 Description of Studies Excluded at Full Text Stage

| Author | Title | Reason for exclusion |
| --- | --- | --- |
| Actrn 2005 | The health-related benefits of country line dancing in community-dwelling older women with functional impairment: a double-blind placebo controlled clinical trial | Not RCT |
| Ades 2005 | Resistance training increases total daily energy expenditure in disabled older women with coronary heart disease | Lack of extractable outcomes |
| Aiello 2004 | Effect of a yearlong, moderate-intensity exercise intervention on the occurrence and severity of menopause symptoms in postmenopausal women | Lack of extractable outcomes |
| Alves 2013 | Creatine Supplementation Associated or Not with Strength Training upon Emotional and Cognitive Measures in Older Women: A Randomized Double-Blind Study | Lack of extractable outcomes |
| Audette 2006 | Tai Chi versus brisk walking in elderly women | Lack of extractable outcomes |
| Bashir 2022 | Effects of Aerobic Exercises on Postmenopausal Depression: Randomized Controlled Trial | Ineligible participants |
| Battaglia 2016 | Effects of an adapted physical activity program on psychophysical health in elderly women | Lack of extractable outcomes |
| Bravo 1996 | Impact of a 12-Month Exercise Program on the Physical and Psychological Health of Osteopenic Women | Lack of extractable outcomes |
| Devereux 2005 | Effects of a water-based program on women 65 years and over: A randomised controlled trial | Lack of extractable outcomes |
| Elavsky 2007 | Physical Activity and Mental Health Outcomes During Menopause: A Randomized Controlled Trial | Ineligible participants |
| Eskiyurt 2019 | HE ROLE OF SUPERVISED EXERCISES ON QUAL ITY OF LIFE AND DEPRESSIVE SYMPTOMS IN  POSTMENOPAUSAL WOMEN | Not RCT |
| Esmaeilzadeh 2013 | Effects of High-impact Exercises on Bone Mineral Density, Bone Turnover Marker, Quality of Life and Depressive Symptoms in Postmenopausal Women: A Randomized Controlled Trial. | Not RCT |
| Eyigor 2009 | A randomized controlled trial of Turkish folklore dance on the physical performance, balance, depression and quality of life in older women | Lack of extractable outcomes |
| R. B. R. g27zn 2012 | Effect of the physical training in community-dwelling older women: randomized clinical assay | Full-text no available |
| Gary2004 | Home-based exercise improves functional performance and quality of life in women with diastolic heart failure | Lack of extractable outcomes |
| Hanachi 2007 | The Effect of Soymilk on Alkaline Phosphatase, Total Antioxidant Levels, and Vasomotor Symptoms in Menopause Women | Inappropriate intervention |
| Hansen 2023 | The effect of aerobic exercise training on asthma control in postmenopausal women (ATOM): a randomized controlled pilot study | Lack of extractable outcomes |
| Huang 2023 | THE LESSENING INCONTINENCE WITH LOW-IMPACT ACTIVITY (LILA) TRIAL: a MULTISITE RANDOMIZED TRIAL OF A PELVIC YOGA VERSUS PHYSICAL CONDITIONING PROGRAM IN MIDLIFE AND OLDER WOMEN WITH URINARY INCONTINENCE | Full-text no available |
| Irct2012112810493N 2013 | Pilates exercise and its effect on falls in elderly women | Full-text no available |
| Irct2014020616501N 2014 | Walking with pedometer, Depression, General health in postmenopausal women | Full-text no available |
| Irct2014042017344N 2014 | Pilates exercise and acupressure effect on anxiety and sleep quality in postmenopausal women | Full-text no available |
| Irct2015020420955N 2015 | The effect of Tai chi chuan on Depression and Anxiety level among elderly women | Full-text no available |
| Irct2015072923408N 2016 | Effect of Tai Chi Exercise on the Stress of Elderly Women  With Hypertension | Not RCT |
| Irct2015122425685N 2016 | The effect of self management program on elderly women with rheumatoid arthritis | Full-text no available |
| Irct20200222046579N 2020 | The effect of physical activity training program on depression and anxiety in Postmenopausal Women | Full-text no available |
| Jin 2019 | A Long-Term Exercise Intervention Reduces Depressive Symptoms in Older  Korean Women | Not RCT |
| Jorge 2016 | Hatha Yoga practice decreases menopause symptoms and improves quality of life: A randomized controlled trial | Lack of extractable outcomes |
| Khalili 2022 | The effect of group walking program on social physique anxiety and the risk of eating disorders in aged women: A Randomized Clinical Trial study | Full-text no available |
| Kline 2016 | Doseeresponse effects of exercise training on the subjective sleep quality of postmenopausal women: exploratory analyses of a randomised controlled trial | Lack of extractable outcomes |
| Lee 2011 | Effects of exercise therapy on cardiac health after menopause: From clinical to bench evidence | Not RCT |
| Lee 2021 | Effects of 16 Weeks of Taekwondo Training on the Cerebral Blood Flow Velocity, Circulating Neurotransmitters, and Subjective Well-Being of Obese Postmenopausal Women | Lack of extractable outcomes |
| iposcki 2019 | Influence of a Pilates exercise program on the quality of life of sedentary elderly people: A randomized clinical trial | Lack of extractable outcomes |
| Macfarlane 2005 | EFFECTS OF TAI CHI ON THE PHYSICAL AND PSYCHOLOGICAL WELL-BEING OF CHINESE OLDER WOMEN | Full-text no available |
| Marcu 2015 | ROLE OF PHYSICAL EXERCICE PROGRAM IN PATIENTS WITH OSTEOPOROSIS | Not RCT |
| Martin 2009 | Exercise Dose and Quality of Life A Randomized Controlled Trial | Lack of extractable outcomes |
| Mason 2019 | Eating behaviors and weight loss outcomes in a 12-month randomized trial of diet and/ or exercise intervention in postmenopausal women | Lack of extractable outcomes |
| Moilanen 2012 | Effect of aerobic training on menopausal symptomsVa randomized controlled trial | Ineligible participants |
| Mokhtari 2013 | Effect of Pilates training on some psychological and social factors related to falling in elderly women | Lack of extractable outcomes |
| Moreau 1999 | The eff The effects of walking v ects of walking volume on blood pr olume on blood pressure in hyper e in hypertensive postmenopausal women | Not RCT |
| Moriyama 2008 | A randomized, placebo-controlled trial of the effects of physical exercises and estrogen therapy on health-related quality of life in postmenopausal women | Lack of extractable outcomes |
| Nct 2023 | Clinical Pilates and Aerobic Exercise in Menopause | Full-text no available |
| Nct 2023 | Effects of Progressive Resistance Training Vasomotor Symptoms in Post Menopausal Women | Full-text no available |
| Nct 2023 | The Effect of Laughter Yoga on Self-Care, Quality of Life and Stress Level in Menopause | Full-text no available |
| Nikkhah 2015 | The effect of walking with pedometer on general health of postmenopausal women | Lack of extractable outcomes |
| Pactr 2019 | Effect of Aerobic Exercise on Plasma Oxytocin in Depressed Post-menopausal Women: a Randomized Controlled Trial | Full-text no available |
| Pirzadeh 2017 | The effect of exercise on menopausal symptoms in postmenopausal women | Not RCT |
| Prieto 2015 | RELEVANCIA DE UN PROGRAMA DE EQUILIBRIO EN LA CALIDAD DE VIDA RELACIONADA CON LA SALUD DE MUJERES ADULTAS MAYORES OBESAS | Lack of extractable outcomes |
| Rashidi 2013 | The effect of eight weeks of aerobic exercise on depression in postmenopausal women | Lack of extractable outcomes |
| Ravari 2021 | The effect of Pilates exercise on the happiness and depression of elderly women: a clinical trial study | Full-text no available |
| Roghani 2012 | The effect of 6-week submaximal training with and without external loading on cardiovascular fitness, balance, cortisol, and lipid profiles in osteoporotic postmenopausal women | Lack of extractable outcomes |
| Salehi 2016 | Effects of 8-weeks combined training (strength and endurance) on serum levels of AGRP, GH and changes in appetite and body mass in postmenopausal women | Lack of extractable outcomes |
| Shen 2010 | Green tea polyphenols supplementation and Tai Chi exercise for postmenopausal osteopenic women: safety and quality of life report | Lack of extractable outcomes |
| Son 2006 | The Effect of the Walking Exercise on Physiological index, Physical Fitness, Self Esteem, Depression and Life Satisfaction in the Institutionalized Elderly Women | Lack of extractable outcomes |
| Susanti 2022 | Effects of yoga on menopausal symptoms and sleep quality across menopause statuses: A randomized controlled trial | Ineligible participants |
| Vedovelli 2017 | Multimodal physical activity increases brain-derived neurotrophic factor levels and improves cognition in institutionalized older women | Not RCT |
| Velez-Toral 2017 | Improvements in health-related quality of life, cardio-metabolic health and fitness in postmenopausal women after an exercise plus health promotion intervention: a randomized controlled trial | Lack of extractable outcomes |
| Xi 2017 | Effect of health education combining diet and exercise supervision in Chinese women with perimenopausal symptoms: a randomized controlled trial | Ineligible participants |
| Yu 2015 | Effects of a Physical Activity Program using Exergame with Elderly Women | Lack of extractable outcomes |
| asbury2006 | The importance of continued exercise participation in quality of life and psychological well-being in previously inactive postmenopausal women: a pilot study | Lack of proper control |
| Blain2017 | EFFECT OF A 6-MONTH BRISK WALKING PROGRAM ON WALKING ENDURANCE IN SEDENTARY AND PHYSICALLY DECONDITIONED WOMEN AGED 60 OR OLDER: A RANDOMIZED TRIAL | Not RCT |
| Lin 2009 | 运动处方干预对老年女性身心健康的影响 | Not RCT |
| Wang 2016 | 广场舞对老年女性生活质量的影响 | Lack of extractable outcomes |
| Ruan 2015 | 规律性瑜伽运动对老年女性生活质量及健康体能的影响 | Lack of extractable outcomes |
| Zheng 2021 | 简化太极拳锻炼对改善老年女性生活质量的作用（Influence of simplified Taijiquan exercise on improving life quality of old women） | Not RCT |
| Mo 2016 | 健身气功太极养生杖锻炼对老年女性心境状态与心理健康的影响 | Lack of extractable outcomes |
| Ma 2015 | 妇女卵巢功能衰退过程中骨密度的变化及健步走运动对绝经过渡期及绝经早期妇女骨密度的影响 | Not RCT |
| Liu 2022 | 12 周弹力带抗阻训练对老年女性下肢肌力、步行功能和认知功能的影响 | Lack of extractable outcomes |
| Wang 2022 | 负重太极拳运动对绝经后骨质疏松症患者平衡及运动功能的影响 | Inappropriate intervention |

| Supplemental Material 6 Study Characteristics Table 10 Study characteristics | | | | | | | | | |
| --- | --- | --- | --- | --- | --- | --- | --- | --- | --- |
| Study | | Participants | | | Interventions | | | | Outcome  Measured |
| Author (year) | Country | Sample size (drop) | Age (SD) | Health  Status | Intervention Program | Duration | Frequency | Length |  |
| Abedi2015 | Iran | EG：53;9.4%  CG:53;7.5% | EG: 52.4±3.8  CG: 53±4.1 | mild to moderate depression | EG:pedometer-based walking  CG:usual care | NR | NR | 12wk | HADS |
| Afonso 2012 | Brazil | EG1: 21;33.3%  EG2:24;37.4%  CG:16;6.2% | ALL:50-65 | insomnia | EG1: passive stretching  EG2:yoga  CG:usual care | 1 hour/day | 2 days/wk | 16wk | BDI、BAI |
| Agil2010 | Turkey | EG1:21;14.3% EG2:21;14.3% | EG1:52.4±4.7  EG2:52.6±3.5 | healthy | EG1: supervised aerobic exercise  EG2: supervised resistance exercise | EG1:40-45minutes/day  EG2:NR | 3days/wk | 8wk | BDI/SCL-90 |
| Aibar-Almazán 2019 | Spain | EG:55;0%  CG:55;5.4% | EG:69.98±7.83  CG:66.79±10.14 | healthy | EG: Pilates-based exercise CG:usual care | 1 hour/day | 2 days/wk | 12wk | HADS |
| Bernard 2015 | France | EG:61;13.1%  CG:60;8.3% | EG:65.46±4.37  CG:65.5±4.03 | healthy | EG:supervised and home-based walking  CG:usual care | 40 minutes/day | 3 days/wk | 24wk | BDI |
| Blumenthal1991 | USA | CG:18:NR  EG1:16;NR EG2:17;NR | ALL:67.7±5.1 | sedentary | EG1: aerobic exercise  EG2: yoga  CG: usual care | 1 hour | EG1: 3 days/wk  EG2: 2 days/wk | 16wk | CES-D/STAI |
| Bowen 2006 | USA | EG1:86;0%  EG2:87;1.1% | ALL:50-75 | Sedentary healthy | EG:home-based aerobic exercise  CG:stretching training | 45 minutes/day | 5 days/wk | 12months | BSI |
| Carcelén-Fraile 2022 | Spain | EG:63;9.5%  CG:62;3.2% | EG:69.70±6.15  CG:69.75±6.76 | healthy | EG:Qigong  CG:usual care | 1 hour/day | 2 days/wk | 12wk | HADS |
| Curi 2018 | Brazil | EG:33;6.0%  CG:31;3.2% | EG:64.25±0.14  CG:63.75±0.08 | healthy | EG:supervised Pilates  CG:usual care | 1hour/day | 2 days/wk | 16wk | GHQ-12 |
| Farzane 2022 | Iran | EG:21;28.6%  CG:17;29.4% | EG:61.47±1.55 CG:62.00±1.4 | healthy | EG:Pilates  CG: usual care | 75minutes/day | 3 days/wk | 8wk | HADS |
| Gary 2007 | Atlanta | EG:13;0%  CG:10;0% | ALL:68±12 | Diastolic Heart Failure | EG: home-based walking  CG: health education | NR | 3 days/wk | 12wk | GDS |
| Gusi 2008 | Spain | EG:64;14.1%  CG:63;19.0% | EG:71±5  CG:74±6 | moderate depression or overweight | EG:supervised walking-based exercise  CG:routine care and a recom-mendation of physical activity | 50minutes/day | 3 days/wk | 24wk | GDS/STAI |
| Gutiérrez 2012 | Spain | EG:30;10%  CG:30;0% | ALL:60-70 | suffered mood problem | EG:multicomponent exercise  CG:usual care | 50-60 minutes/day | 3day/wk | 24wk | GDS/HRSA |
| Hu 2017 | China | EG:46;13.0%  CG:45;11.1% | EG:52.60±4.12  CG:54.15±2.32 | healthy | EG: supervised walking  CG: usual care | 1 hour/day | 3 days/wk | 16wk | BDI |
| Imayama 2011 | USA | EG:117;10.3%  CG:87;8.0% | EG:58.1±5.0  CG:57.4±4.4 | overweight/obese | EG:supervised aerobic exercise  CG:usual care | 45 minutes | 5 days/wk | 48wk | BSI-18 |
| Innes 2012 | USA | EG:10;0%  CG:10;0% | EG:58.40±6.32  CG:58.90±9.11 | overweight/restless legs syndrome | EG:yoga  CG:educational film | 90-minutes/day | 2 days/wk | 8wk | POMS |
| Kim 2019 | Korea | EG:12;8.3%  CG:13;23.1% | EG:76.10±3.85  CG:76.40±3.27 | healthy | EG:strength training  CG:usual care | 50-80minutes/day | 3 days/wk | 24wk | SGDS-K |
| Sen2019 | Turkey | EG:19;15.8%  CG:20;10% | EG:53.1±4.4 CG:54.5±6.0 | osteoporosis | EG: multicomponent exercises  CG: usual care | 20-60minutes/day | 3 days/wk | 24wk | BDI |
| Liu 2016 | China | EG:32;3.1%  CG:32;3.1% | EG:66.3±2.7  CG:65.8±3.2 | healthy | EG: Tai Chi  CG: usual care | 60minutes/day | 5 days/wk | 16 wk | POMS/SCL-90 |
| Ma2016 | China | EG:40;5%  CG:40;0% | EG:60.9±5.1  CG:60.2±4.1 | healthy | EG:Qigong  CG:usual care | 1 hour/day | 3 days/wk | 20wk | POMS/SAS |
| Pereira 2013 | Brazil | EG1:229;21.0%  EG2:222;24.8% | EG1:71.03±4.8  EG2:70.33±4.5 | inactive | EG1: supervised strengthening  EG2: aerobic exercise | 1 hour/day | 3 days/wk | 10wk | GDS |
| Pinheiro 2020 | Brazil | EG:20;50%  CG:20;45% | EG:79±7.66  CG:81.7±5.95 | sarcopenia | EG:supervised progressive resistance training  CG:usual care | NR | 2 days/wk | 12wk | GDS |
| Sen2019 | Turkey | EG:19;15.8%  CG:20;10% | EG:53.1±4.4 CON:54.5±6.0 | osteoporosis | EG:supervised aerobic, resistance and streching exercise  CG:usual care | 20-60min | 3 days/wk | 24wk | BDI |
| Shahidi 2011 | Iran | EG1:23;13.0%  EG2:23;13.0% CG:24;16.7% | CG: 68.4±6.3 EG1:65.5±4.8 EG2:65.7±4.2 | depressed | EG1: laughter yoga  EG2: exercise therapy  CG:usual care | EG1:NR  EG2:30minutes/day | NR | NR | GDS |
| Song 2022 | China | EG:20;0%  CG:20;0% | EG:64.15±8.56  CG:64.15±8.56 | knee osteoarthritis | EG:Tai Chi  CG:wellness education | 60minutes/day | 3 days/wk | 12 wk | SDS/SAS |
| Soori 2022 | Iran | EG1:25; 0% EG2:25;0%  CG:25;0% | EG1:62.48±2.87 EG2:62.66±1.68  CG:63.80±3.35 | inactive | EG1: aerobic exercise  EG2: Pilates  CG:usual care | 40 minutes/day | EG1:3 days/wk  EG2:NR | 3wk | GHQ |
| Williams 1997 | Malaysia | EG :94;24.5%  CG:93;16.1% | EG :71.8±5.6  CG:71.6±5.2 | Osteoarathritis;High blood pressure;Heart disease | EG:aerobic exercises and strengthening exercises  CG:usual care | 1 hour/day | 2 days/wk | 42wk | DASS |

*Note:* SD: standard deviation; EG: exercise group; CG: control group; NR: not report; HADS: Hospital Anxiety and Depression Scale; BDI: Beck Depression Inventory; BAI: Beck Anxiety Inventory; GDS: Geriatric Depression Scale; CES-D: Center for Epidemiologic Studies Depression Scale; STAI: State-Trait Anxiety Inventory; SCL-90: Symptom Checklist-90; POMS: Profile of Mood States; SDS: Self-Rating Depression Scale; SAS: Self-Rating Anxiety Scale; HRSA: Hamilton Rating Scale for Anxiety; DASS: Depression Anxiety Stress Scales; GHQ: General Health Questionnaire.

Supplemental Material 7 The risk of bias for each study

Figure 1 The risk of bias for each study


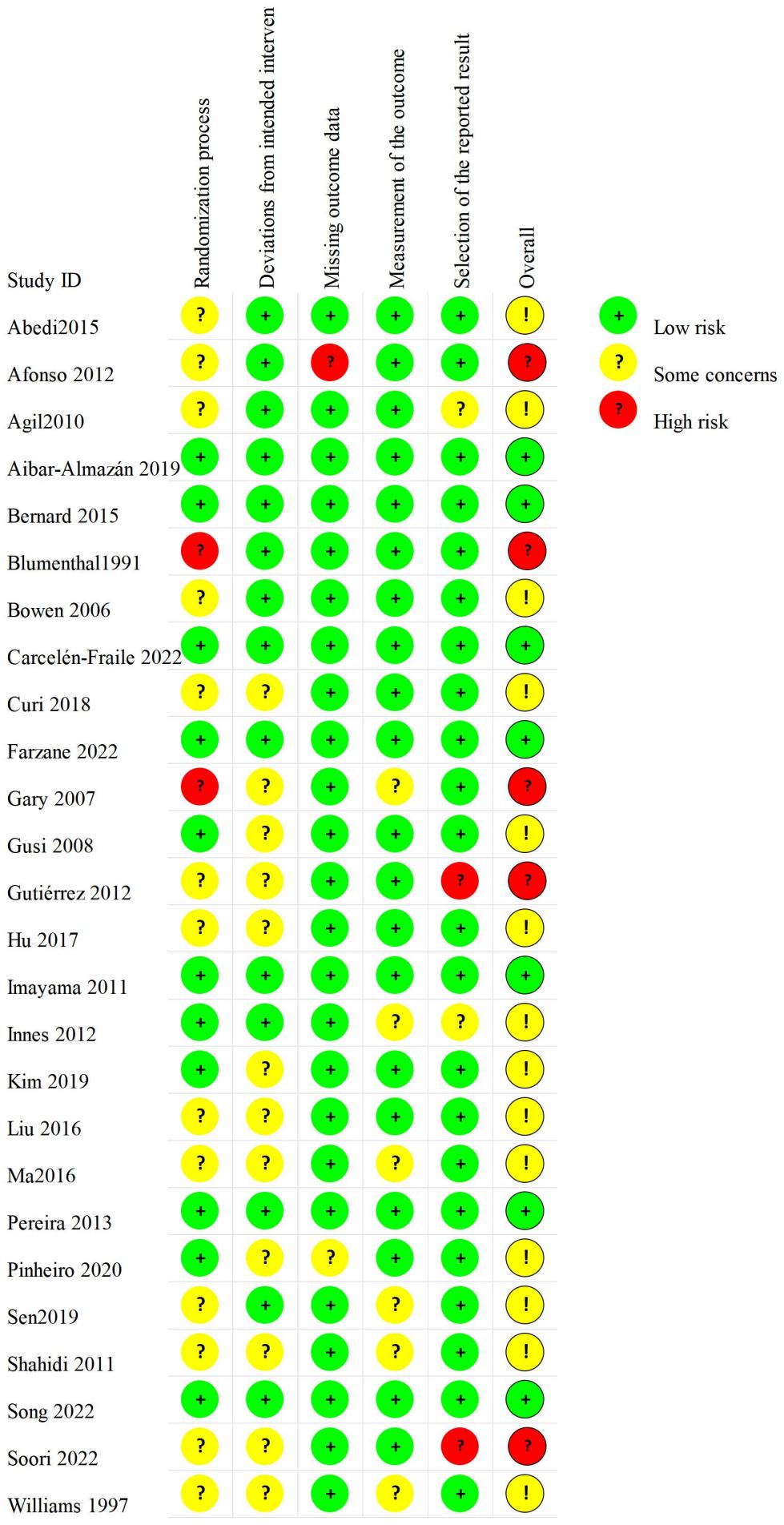


**Supplemental Material 8 Evaluation of the Certainty of evidence**

Table 11 Evaluation of the Certainty of evidence Using CINeMA Framework, mixed evidence

| Comparison | | N | Within-study bias | Reporting bias | Indirectness | Imprecision | Heterogeneity | Incoherence | Confidence rating | Reasons for downgrading |
| --- | --- | --- | --- | --- | --- | --- | --- | --- | --- | --- |
| Mixed evidence | | | | | | | | | | |
| CON vs SE | 1 | | Some concerns | Undetected | No concerns | Major concerns | No concerns | No concerns | Very low | -1^*^ Within-study bias  -2^*^ Imprecision |
| CON vs AE | 9 | | Some concerns | Undetected | No concerns | No concerns | Major concerns | No concerns | Very low | -1^*^ Within-study bias  -2^*^ Heterogeneity |
| CON vs RT | 2 | | Some concerns | Undetected | No concerns | Some concerns | Some concerns | No concerns | Very low | -1^*^ Within-study bias  -1^*^ Imprecision  -1^*^ Heterogeneity |
| CON vs MB | 11 | | Some concerns | Undetected | No concerns | No concerns | No concerns | No concerns | Moderate | -1^*^ Within-study bias |
| CON vs ME | 2 | | Some concerns | Undetected | No concerns | Some concerns | Some concerns | No concerns | Very low | -1^*^ Within-study bias  -1^*^ Imprecision  -1^*^ Heterogeneity |
| SE vs AE | 1 | | Some concerns | Undetected | No concerns | Major concerns | No concerns | No concerns | Very low | -1^*^ Within-study bias  -2^*^ Imprecision |
| SE vs MB | 1 | | Some concerns | Undetected | No concerns | Some concerns | Some concerns | No concerns | Very low | -1^*^ Within-study bias  -1^*^ Imprecision  -1^*^ Heterogeneity |
| AE vs RT | 2 | | Some concerns | Undetected | No concerns | Major concerns | No concerns | No concerns | Very low | -1^*^ Within-study bias  -2^*^ Imprecision |
| AE vs MB | 3 | | Some concerns | Undetected | No concerns | No concerns | Major concerns | No concerns | Very low | -1^*^ Within-study bias  -2^*^ Heterogeneity |
| Indirect evidence | | | | | | | | | | |
| SE vs RT | - | | Some concerns | Undetected | No concerns | Major concerns | No concerns | No concerns | Very low | -1^*^ Within-study bias  -2^*^ Imprecision |
| SE vs ME | - | | Some concerns | Undetected | No concerns | Major concerns | No concerns | No concerns | Very low | -1^*^ Within-study bias  -2^*^ Imprecision |
| AE vs ME | - | | Some concerns | Undetected | No concerns | Major concerns | No concerns | No concerns | Very low | -1^*^ Within-study bias  -2^*^ Imprecision |
| RT vs MB | - | | Some concerns | Undetected | No concerns | Some concerns | Some concerns | No concerns | Very low | -1^*^ Within-study bias  -1^*^ Imprecision  -1^*^ Heterogeneity |
| RT vs ME | - | | Some concerns | Undetected | No concerns | Major concerns | No concerns | No concerns | Very low | -1^*^ Within-study bias  -2^*^ Imprecision |
| MB vs ME | - | | Some concerns | Undetected | No concerns | Major concerns | No concerns | No concerns | Very low | -1^*^ Within-study bias  -2^*^ Imprecision |

*Note:*

CON, control; SE, stretching exercise; AE, aerobic exercise; RT, resistance training; MBE, mind-body exercise; ME, multicomponent exercise.

−1* signifies a one-level downgrading of each relative treatment effect’s confidence.

−2* signifies a two-level downgrading of each relative treatment effect’s confidence.

Supplemental Material 9 The Analysis for Primary Outcomes

9.1 Publication Bias Test on Depression in Pairwise Meta-analysis

9.1.1 Funnel Plot of Exercise Effects on Depression in Pairwise Meta-analysis

**
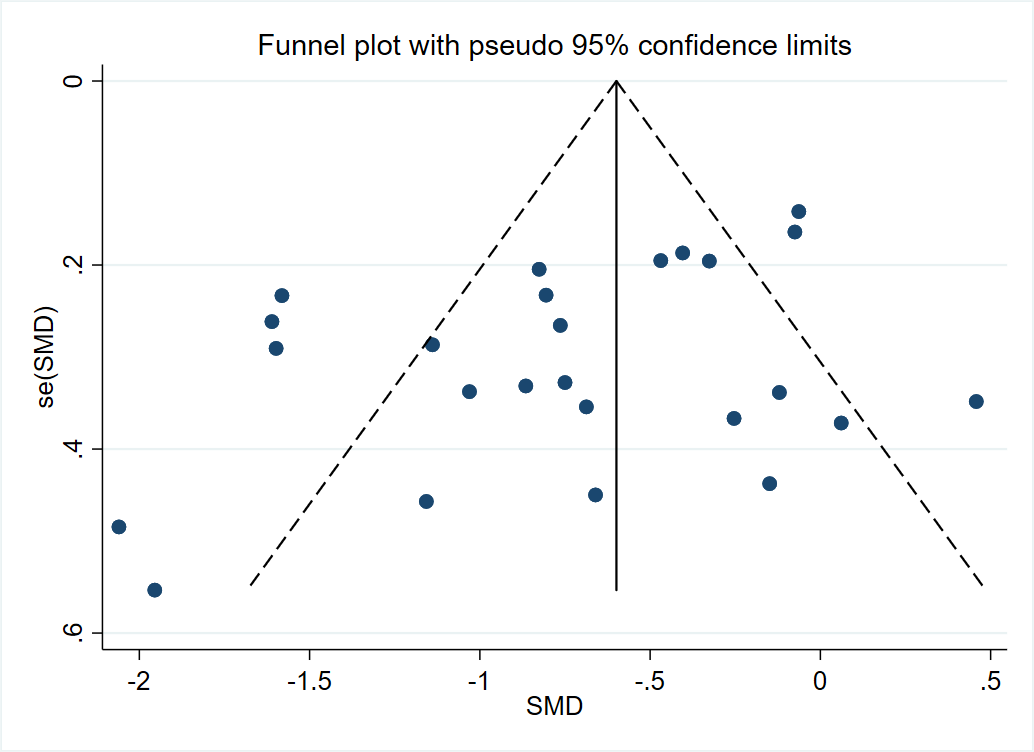
**

Figure 2 Funnel Plot of Exercise Effects on Depression in Postmenopausal Women

**9.1.2 Trim-and-fill Funnel plot of Exercise Effects on Depression in Pairwise Meta-analysis**

Figure 3 Trim-and-fill Funnel plot of Exercise Effects on Depression Levels in Postmenopausal Women

9.2 The Results of the Sensitivity Analysis of Pairwise Meta-analysis

Sensitivity Analysis 1: leave-one-out approach

Figure 4 The results of leave-one-out approach

Sensitivity Analysis 2: Excluding Studies Using Health Education as Control


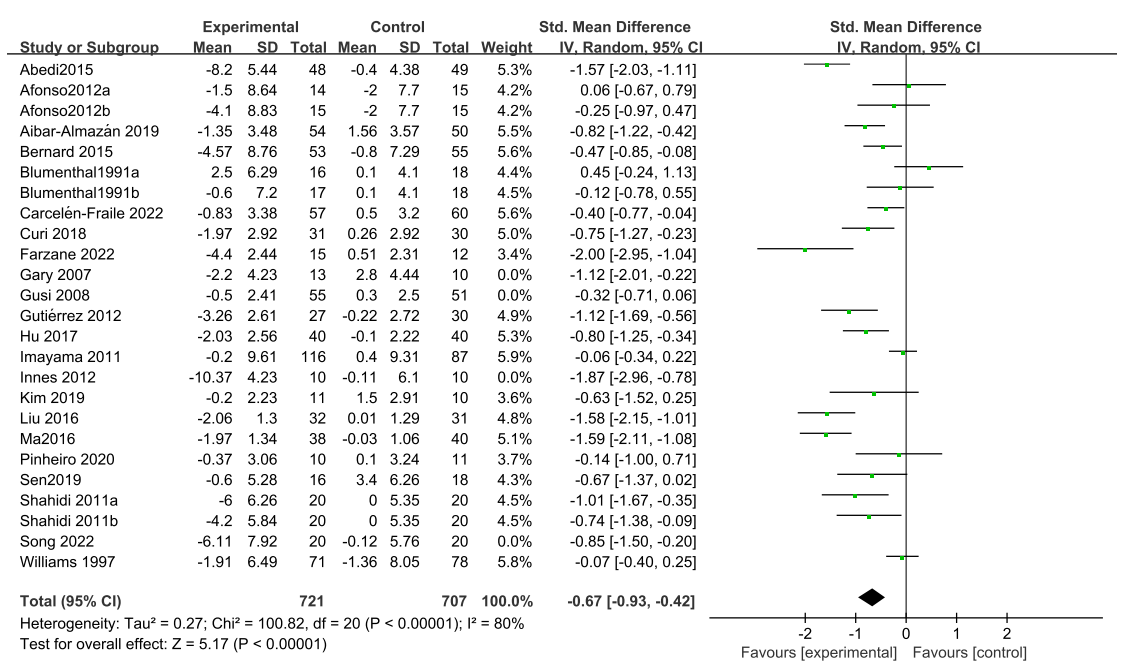


Figure 5 The results of Excluding Studies Using Health Education as Control

Sensitivity Analysis 3: Excluding Studies judged to have an overall high risk of bias


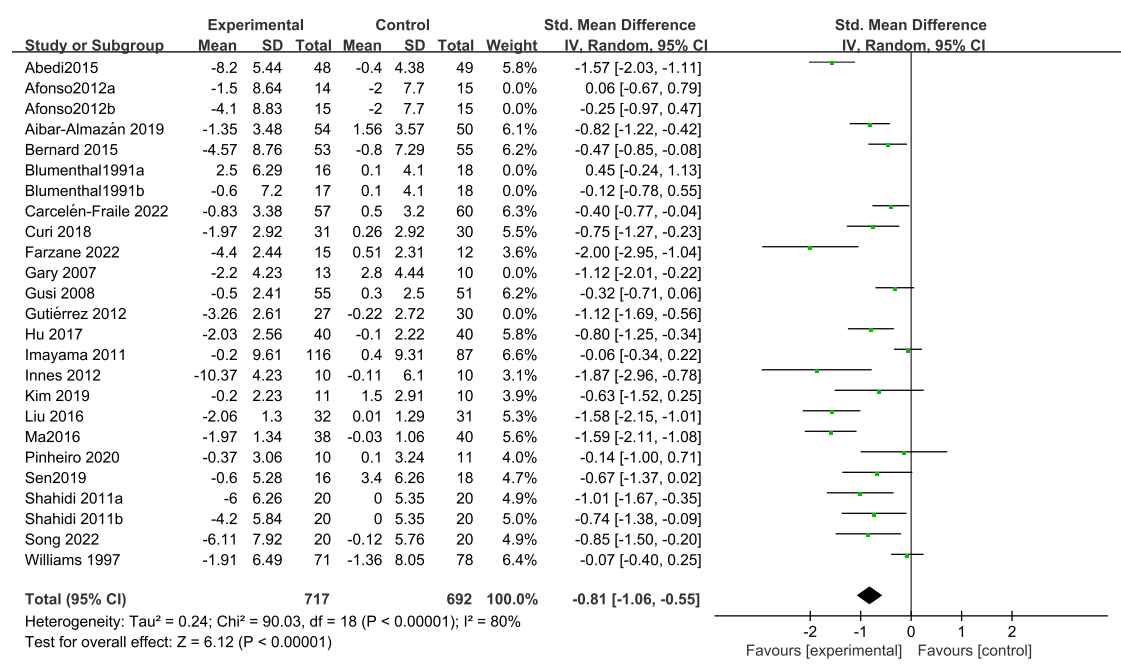


Figure 6 The results of Excluding Studies judged to have an overall high risk of bias

9.3 Local Inconsistency Test for Network Meta-analysis

**9.3.1 The Result of Loop Inconsistency Test**


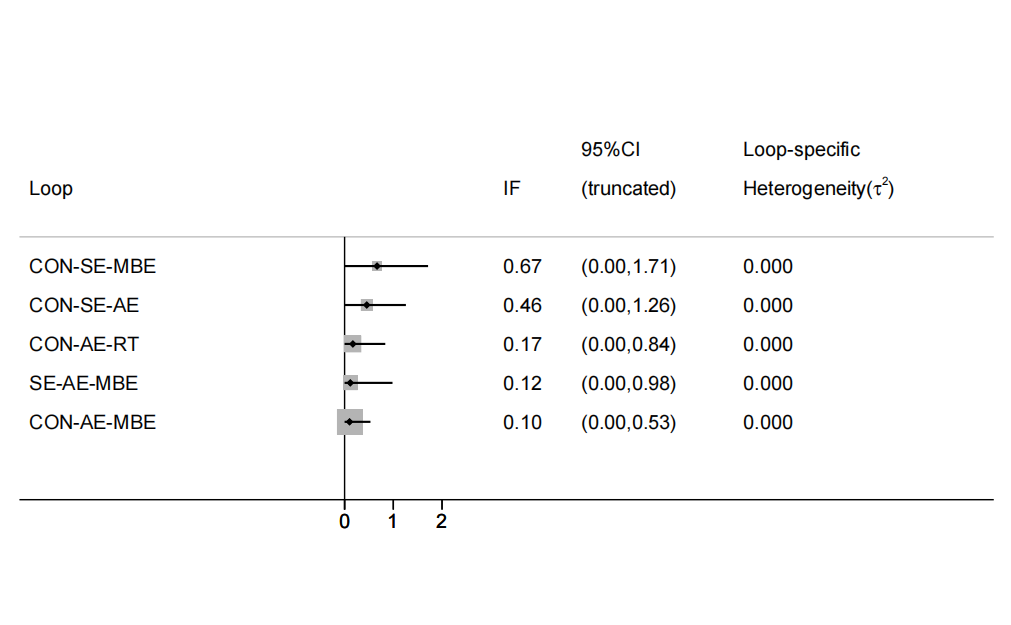


**Figure 7 Loop inconsistency in depression.** CON, control; SE, stretching exercise; AE, aerobic exercise; RT, resistance training; MBE, mind-body exercise.

**9.3.2 The Result of Node-splitting Method**

Table 12 The Result of Node-splitting Method

| Comparison | Direct | | Indirect | | Difference | | *P* |
| --- | --- | --- | --- | --- | --- | --- | --- |
|  | SMD | se | SMD | se | SMD | se |  |
| CON vs SE | 0.062 | 0.570 | -0.697 | 0.444 | 0.759 | 0.721 | 0.292 |
| CON vs AE | -0.563 | 0.182 | -0.624 | 0.331 | 0.061 | 0.378 | 0.872 |
| CON vs RT | -0.384 | 0.442 | -0.560 | 0.387 | 0.175 | 0.588 | 0.766 |
| CON vs MBE | -0.959 | 0.165 | -1.124 | 0.480 | 0.164 | 0.507 | 0.746 |
| SE vs AE | -0.074 | 0.467 | -0.283 | 0.529 | 0.208 | 0.706 | 0.768 |
| SE vs MBE | -0.299 | 0.577 | -0.742 | 0.472 | 0.442 | 0.745 | 0.552 |
| AE vs RT | 0.031 | 0.351 | 0.206 | 0.472 | -0.175 | 0.588 | 0.766 |
| AE vs MBE | -0.354 | 0.314 | -0.429 | 0.255 | 0.076 | 0.404 | 0.852 |

Note: CON, control; SE, stretching exercise; AE, aerobic exercise; RT, resistance training; MBE, mind-body exercise.

9.4 The Depression Rankings for Different Types of Exercise.

Table 12 Comparative Efficacy of Exercise Interventions for Depression

| Exercise | SUCRA (%) | PrBest (%) | Mean rank |
| --- | --- | --- | --- |
| Control | 3.8 | 0.0 | 5.8 |
| Stretching exercise | 40.8 | 5.0 | 4.0 |
| Aerobic exercise | 56.4 | 0.9 | 3.2 |
| Resistance training | 47.6 | 5.1 | 3.6 |
| Mind-body exercise | 94.9 | 78.7 | 1.3 |
| Multicomponent exercise | 56.4 | 10.3 | 3.2 |

9.5 The Results of the Sensitivity Analysis of Network Meta-analysis


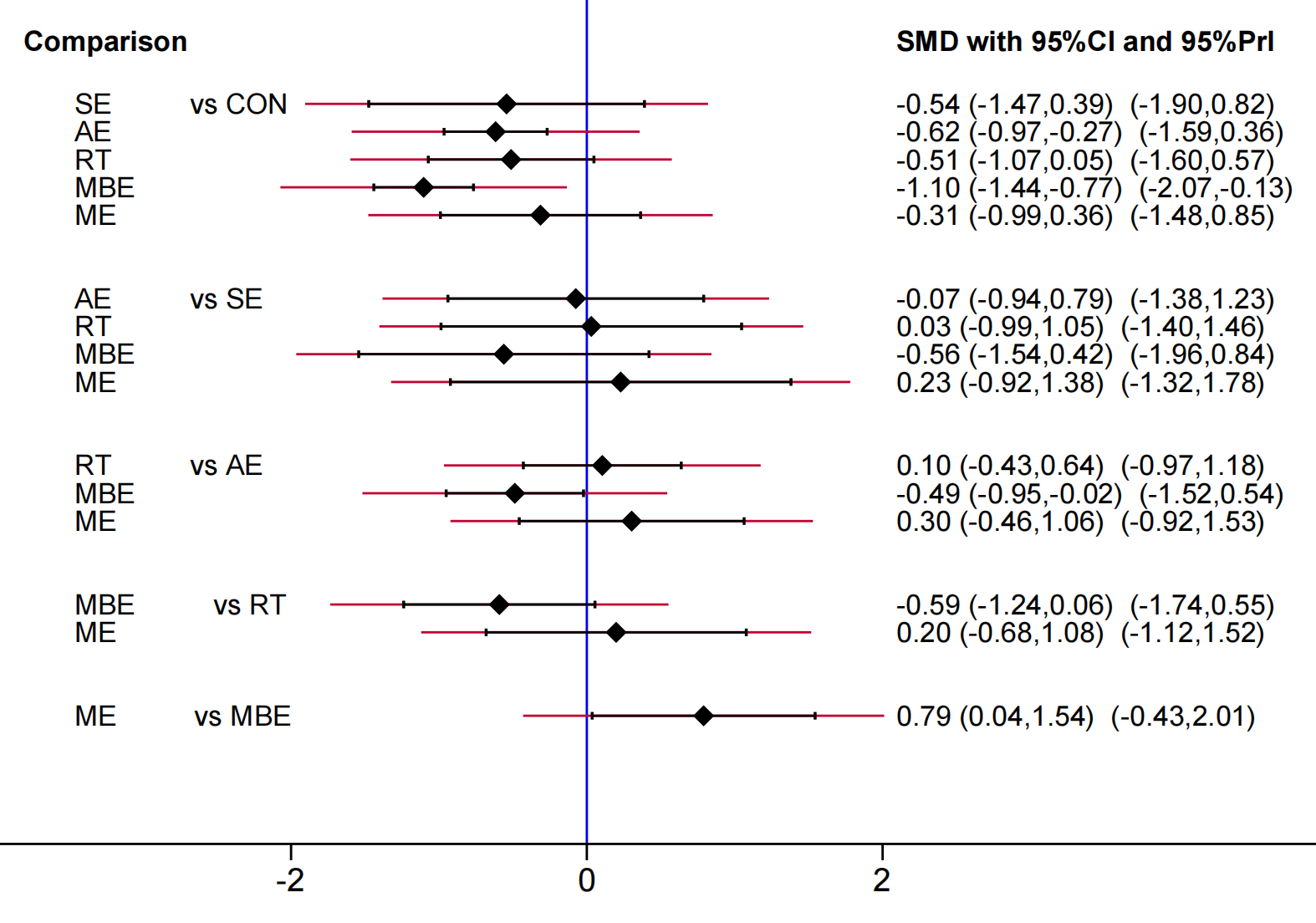


**Figure 8 Predictive interval plot for the depression network.** CON, control; SE, stretching exercise; AE, aerobic exercise; RT, resistance training; MBE, mind-body exercise; ME, multicomponent exercise.

Table 13 Comparative Efficacy of Exercise Interventions for Depression

| Exercise | SUCRA (%) | PrBest (%) | Mean rank |
| --- | --- | --- | --- |
| Control | 7.0 | 0.0 | 5.6 |
| Stretching exercise | 52.0 | 12.8 | 3.4 |
| Aerobic exercise | 60.2 | 0.8 | 3.0 |
| Resistance training | 50.2 | 2.8 | 3.5 |
| Mind-body exercise | 95.7 | 81.8 | 1.2 |
| Multicomponent exercise | 34.9 | 1.8 | 4.3 |

Supplemental Material 10 The Analysis for Anxiety

10.1 Pairwise Meta-analysis for Anxiety

Forest plot：


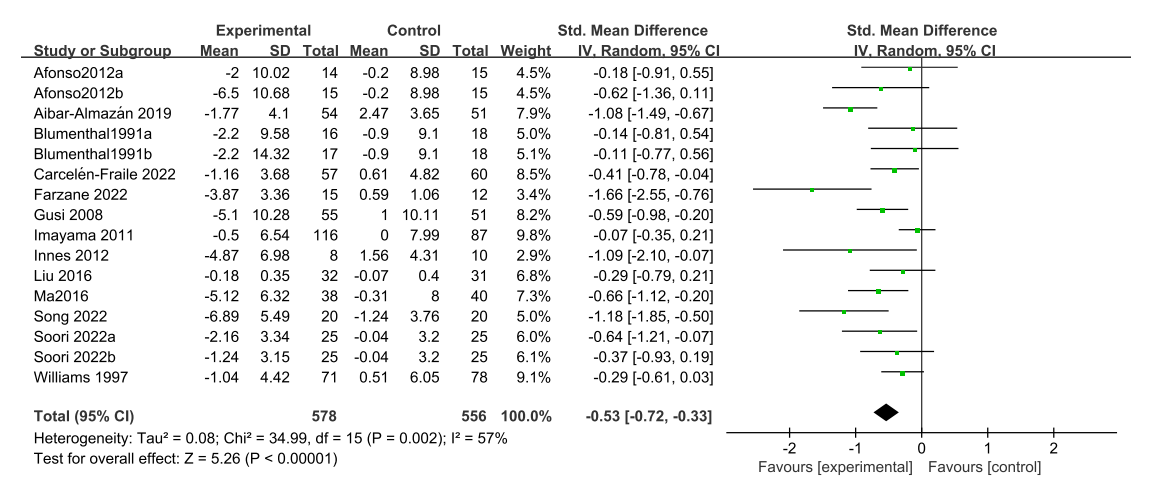


Figure 9 Forest plot of the effects of exercise training on anxiety.

Publication bias test A (Begg’s and Egger’s test)：


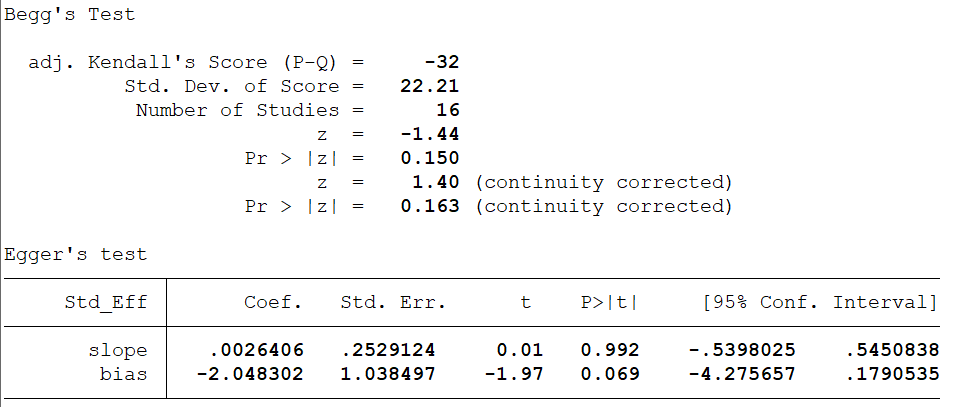


Figure 10 The results of Begg’s and Egger’s test

Publication bias test B (Funnel Plot)


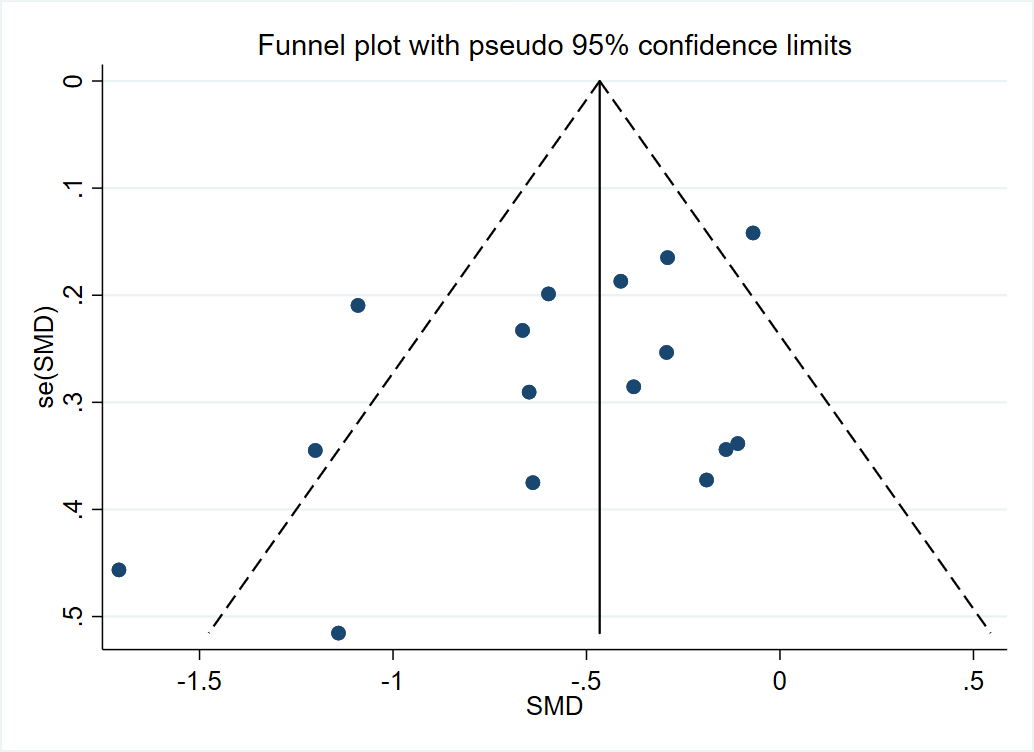


Figure 11 Funnel Plot of Exercise Effects on Anxiety Levels in Postmenopausal Women

10.2 Network Meta-analysis for Anxiety

**
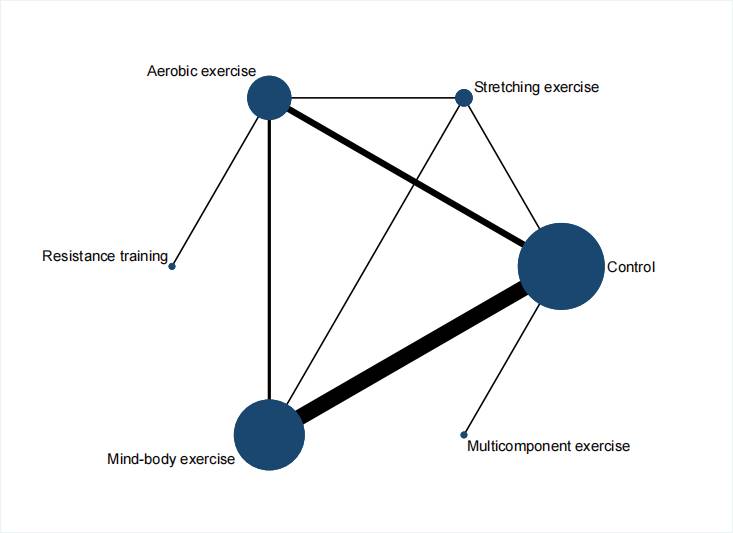
**

Figure 12 Network plot of comparisons for anxiety in the NMA.


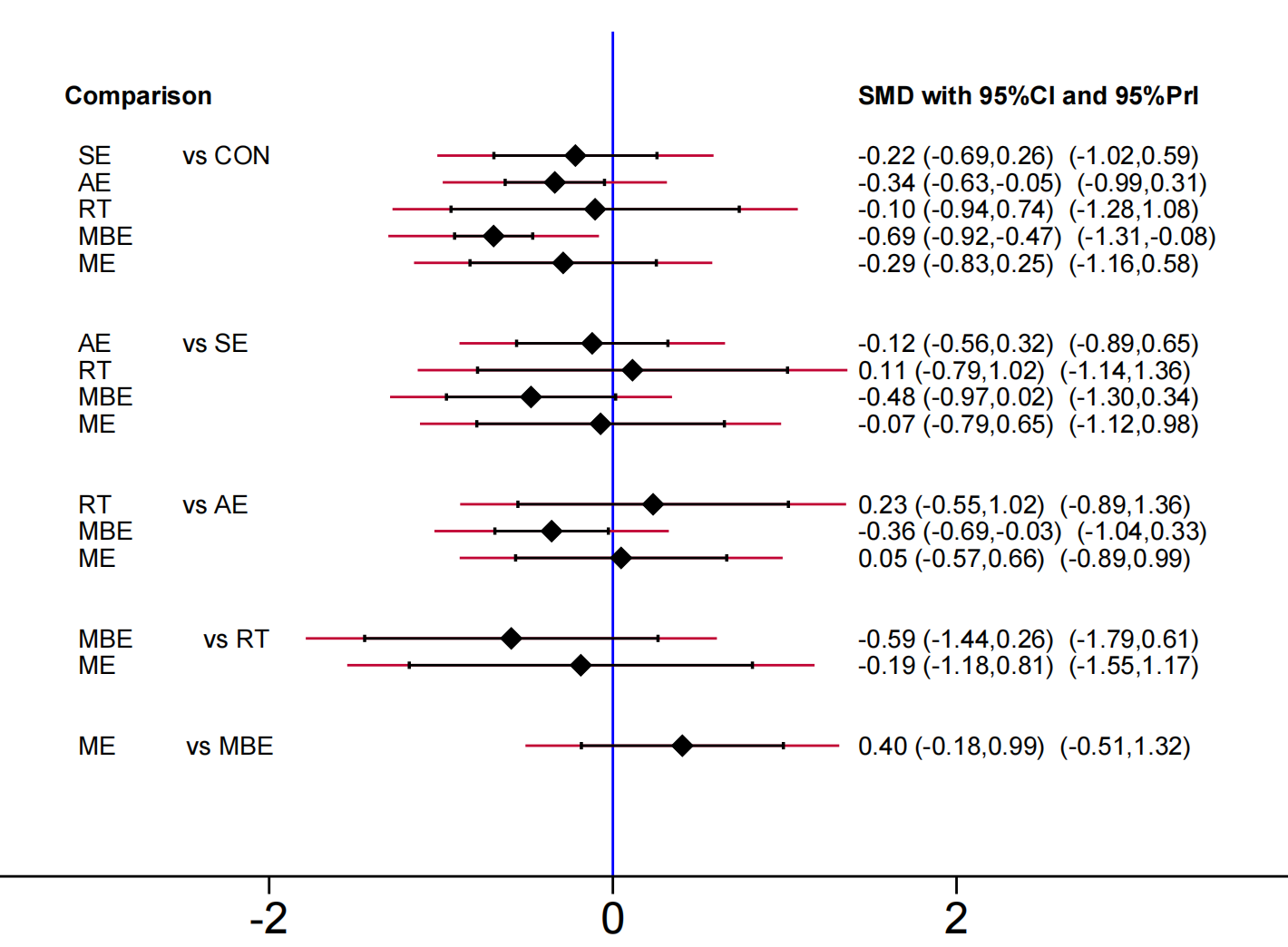


Figure 13 Predictive interval plot for the anxiety network. CON, control; SE, stretching exercise; AE, aerobic exercise; RT, resistance training; MBE, mind-body exercise; ME, multicomponent exercise.

**
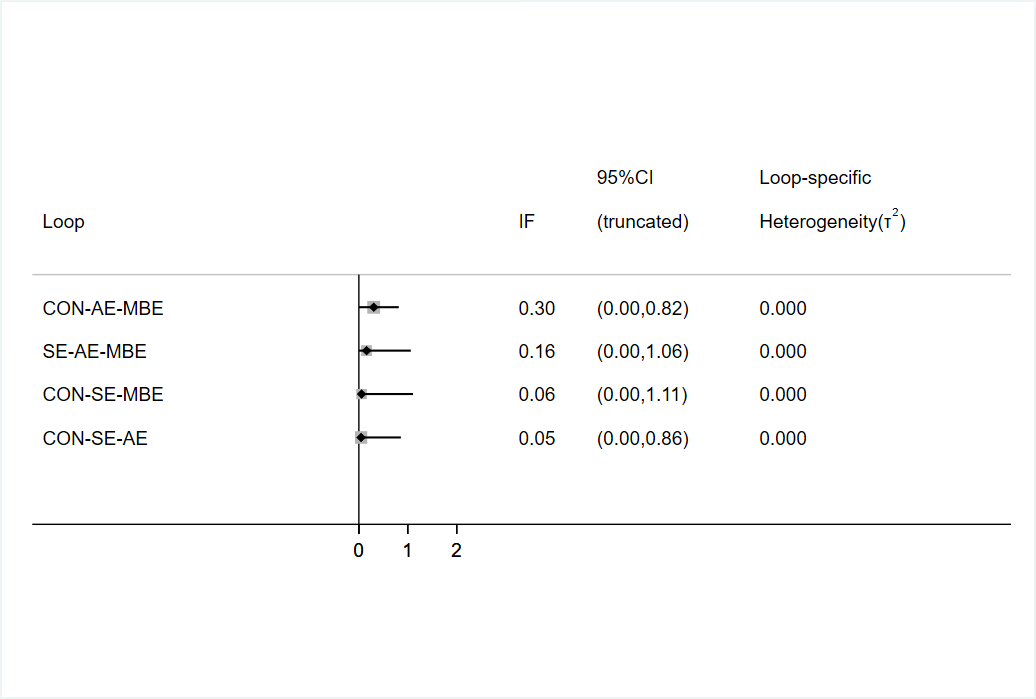
**

Figure 14 Local inconsistency in anxiety. CON, control; SE, stretching exercise; AE, aerobic exercise; MBE, mind-body exercise.


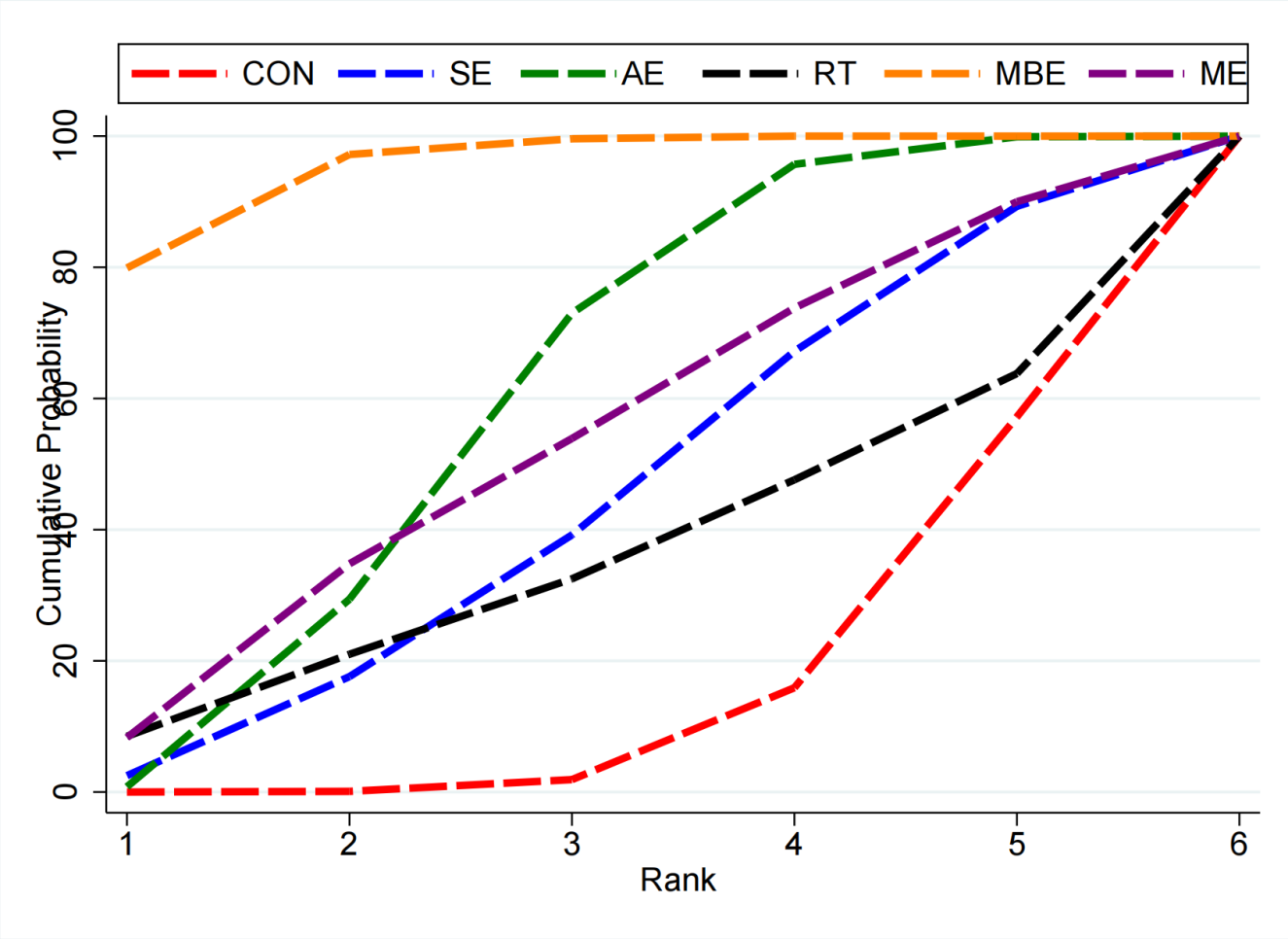


**Figure 15 Cumulative ranking probability plots for anxiety.** CON, control; SE, stretching exercise; AE, aerobic exercise; RT, resistance training; MBE, mind-body exercise; ME, multicomponent exercise.

Table 14 Comparative Efficacy of Exercise Interventions for Anxiety

| Exercise | SUCRA (%) | PrBest (%) | Mean rank |
| --- | --- | --- | --- |
| Control | 15.0 | 0.0 | 5.2 |
| Stretching exercise | 43.1 | 2.5 | 3.8 |
| Aerobic exercise | 59.8 | 0.8 | 3.0 |
| Resistance training | 34.7 | 8.5 | 4.3 |
| Mind-body exercise | 95.4 | 79.9 | 1.2 |
| Multicomponent exercise | 52.1 | 8.3 | 3.4 |
